# Dex-CSDH randomised, placebo-controlled trial of dexamethasone for chronic subdural haematoma: report of the internal pilot phase

**DOI:** 10.1038/s41598-019-42087-z

**Published:** 2019-04-10

**Authors:** Ellie Edlmann, Eric P. Thelin, Karen Caldwell, Carole Turner, Peter Whitfield, Diederik Bulters, Patrick Holton, Nigel Suttner, Kevin Owusu-Agyemang, Yahia Z. Al-Tamimi, Daniel Gatt, Simon Thomson, Ian A. Anderson, Oliver Richards, Monica Gherle, Emma Toman, Dipankar Nandi, Phillip Kane, Beatrice Pantaleo, Carol Davis-Wilkie, Silvia Tarantino, Garry Barton, Hani J. Marcus, Aswin Chari, Antonio Belli, Simon Bond, Rafael Gafoor, Sarah Dawson, Lynne Whitehead, Paul Brennan, Ian Wilkinson, Angelos G Kolias, Peter J. A. Hutchinson, Dex-CSDH trial collaborative and BNTRC collaborative., Khaled Badran, Ian Coulter, Mathew J. Gallagher, Florence R. A. Hogg, Catherine Pringle, Adam Razak, Hamzah Soleiman, Rory Piper, Marian Vintu, Adam Wahba, Anthony Wiggins, Kamal Makram Yakoub, Malik Zaben, Ardalan Zolnourian, Peter Bodkin, Emanuel Cirstea, Giles Critchley, Charlotte Eglinton, Louise Finlay, Daniela Georgieva, Nihal Gurusinghe, Nikolaos Haliasos, Damian Holliman, Kismet Hossain-Ibrahim, Masood Hussain, Jothy Kandasamy, Mary Kambafwile, Ravindra Nannapaneni, Laura Ortiz-Ruiz de Gordoa, Marios C Papadopoulos, Dimitris Paraskevopoulos, Jash Patel, Kuskoor Seetharam Manjunath Prasad, Nikolaos Tzerakis

**Affiliations:** 1 Department of Clinical Neuroscience, University of Cambridge, Cambridge Biomedical Campus, Cambridge, CB2 0QQ UK; 2 Division of Neurosurgery, Box 167, Addenbrooke’s Hospital, Cambridge, CB2 0QQ UK; 3 Department of Clinical Neuroscience, Karolinska Institutet, Stockholm, Sweden; 4 Southwest Neurosurgical Centre, Plymouth University Hospitals NHS trust, Plymouth, PL6 8DH UK; 5 Wessex Neurological Centre, University Hospital Southampton NHS Foundation Trust, Tremona Rd, Southampton, Hampshire, SO16 6YD UK; 6 Institute of Neurosciences, Queen Elizabeth University Hospital, 1345 Govan Road, Glasgow, G51 4TF UK; 7 Department of Neurosurgery, Sheffield Teaching Hospitals NHS Trust, Royal Hallamshire Hospital, Glossop Road, Sheffield, S10 2JF UK; 8 Department of Neurosurgery, Leeds General Infirmary, Great George Street, Leeds, LS1 3EX UK; 9 Department of Neurosurgery, University Hospital Birmingham NHS Foundation Trust, Edgbaston, Birmingham, B15 2WB UK; 10 Department of Neurosurgery, Imperial College Healthcare NHS Trust, Fulham Palace Road, London, W6 8RF UK; 11 Department of Neurosurgery, The James Cook University Hospital, Marton Road, Middlesbrough, TS4 3BW UK; 12 Cambridge Clinical Trials Unit, Box 401, Cambridge Biomedical Campus, Cambridge, CB2 0QQ UK; 13 Norwich Medical School, University of East Anglia, Norwich, NR4 7TJ UK; 14 Department of Neurosurgery, Barts Health NHS trust, Whitechapel Road, London, E1 1BB UK; 15 NIHR Surgical Reconstruction and Microbiology Research Centre & University Hospitals Birmingham NHS Foundation Trust, School of Clinical and Experimental Medicine, University of Birmingham, Institute of Biomedical Research (West), Room WX 2.61, Edgbaston, Birmingham, B15 2TT UK; 16 MRC Biostatistics Unit, Robinson Way, Cambridge Biomedical Campus, Cambridge, CB2 0SR UK; 17 Clinical Trials Pharmacy, Addenbrooke’s Hospital, Cambridge Biomedical Campus, Cambridge, CB2 0QQ UK; 18 Department of Clinical Neurosciences, Western General Hospitals NHS Trust, Crewe Road, Edinburgh, EH4 2XU UK; 19 Department of Neurosurgery, NHS Tayside, Ninewells Hospital, Dundee, DD1 9SY UK; 20 Regional Neurosciences Centre, The Newcastle upon Tyne Hospitals NHS foundation trust, Royal Victoria Infirmary, Queen Victoria Road, Newcastle Upon Tyne, NE1 4LP UK; 21 Department of Neurosurgery, Atkinson Morley Wing, St.George’s Hospital, Blackshaw road, London, SW17 0QT UK; 22 Department of Neurosurgery, Royal Preston Hospital, Sharoe Green Lane North, Fulwood, Preston, Lancashire PR2 9HT UK; 23 Department of Neurosurgery, Hull Royal Infirmary, Anlaby Road, Hull, HU3 2JZ UK; 24 Department of Neurological Surgery, John Radcliff Hospital, Headley Way, Headington, Oxford, OX3 9DU UK; 25 Department of Neurosurgery, Royal Sussex County Hospital, Eastern Road, Brighton, East Sussex BN2 5BE UK; 26 Department of Neurosurgery, Aberdeen Royal Infirmary, Forsterhill, Aberdeen, AB25 2ZN UK; 27 Department of Neurosciences, University Hospital of Wales, Heath Park, Cardiff, CF14 4XW UK; 28 Department of Neurosurgery, The James Cook University Hospital, Marton Road, Middlesbrough, TS4 3BW UK; 29 Department of Neurosurgery, Queen’s Hospital, Rom Valley Way, Romford, RM7 0AG UK; 30 Department of Neurosurgery, Royal Stoke University Hospitals, Newcastle Road, Stoke-on-Trent, ST4 6QG UK

## Abstract

The Dex-CSDH trial is a randomised, double-blind, placebo-controlled trial of dexamethasone for patients with a symptomatic chronic subdural haematoma. The trial commenced with an internal pilot, whose primary objective was to assess the feasibility of multi-centre recruitment. Primary outcome data collection and safety were also assessed, whilst maintaining blinding. We aimed to recruit 100 patients from United Kingdom Neurosurgical Units within 12 months. Trial participants were randomised to a 2-week course of dexamethasone or placebo in addition to receiving standard care (which could include surgery). The primary outcome measure of the trial is the modified Rankin Scale at 6 months. This pilot recruited ahead of target; 100 patients were recruited within nine months of commencement. 47% of screened patients consented to recruitment. The primary outcome measure was collected in 98% of patients. No safety concerns were raised by the independent data monitoring and ethics committee and only five patients were withdrawn from drug treatment. Pilot trial data can inform on the design and resource provision for substantive trials. This internal pilot was successful in determining recruitment feasibility. Excellent follow-up rates were achieved and exploratory outcome measures were added to increase the scientific value of the trial.

## Introduction

In a chronic subdural haematoma (CSDH), blood and fluid collect in the subdural space overlying the brain. It primarily affects elderly patients, many of whom have experienced a head trauma within the preceding weeks^1^. Recent literature suggests a critical role for inflammation in causing fluid and blood exudation from neovascularised subdural membranes^2^. The application of steroids with their potent anti-inflammatory effect is therefore logical, and has shown potential in smaller, non-randomised studies^3–9^.

A multi-institutional group of clinicians and academics in the United Kingdom (UK) designed the Dex-CSDH trial to address a gap in evidence. The trial aims to investigate whether dexamethasone can improve the 6-month functional outcome of patients with symptomatic CSDH by reducing the rate of surgical intervention and recurrence. This paper describes the feasibility phase (internal pilot) of the Dex-CSDH trial.

**Figure 1:**
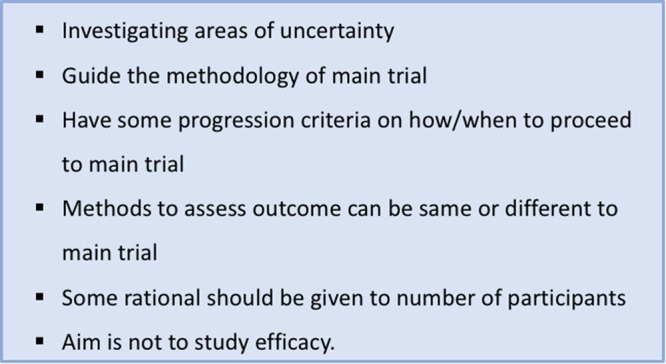
relevant methodological considerations in a pilot trial^10^.

The Consolidated Standards of Reporting Trials (CONSORT) recommendations have recently been extended to include pilot trials, all of which are addressed in this pilot (Fig. 1)^10^, alongside the standard CONSORT checklist (see supplementary information). The data collected during internal pilots can be used in the final substantive trial analysis, and thus has remained blinded, but is still helpful for informing on the design of future pilot trials^11^.

## Primary Pilot Trial Objective

The primary objective of the Dex-CSDH pilot Trial is to assess recruitment feasibility across UK neurosurgical units (NSU). Several factors may influence enrolment of patients and the following were considered in design of this trial;

1. The UK has helped take a lead on improving research in neurosurgery, being 3^rd^ in the world for publishing neurosurgery randomised control trials (RCT) between 2000-2014^12^. This has been a challenge due to historically low levels of neurosurgical RCTs which can result in deficient research infrastructure, training and experience^13^. Of 64 neurosurgical RCTs registered between 2000–2012, 17 (26.6%) were discontinued early, mainly because of insufficient patient recruitment^14^. To avoid this, focus on engagement of local research teams to maximise patient recruitment and retention is essential.
2. Head trauma confers specific challenges to recruitment. The need for emergency intervention limits the time available for clinicians and patients to consider enrolment into clinical trials. Patients often lack capacity and the non-availability of a legal representative (e.g. next-of-kin (NOK)) can impede recruitment. In 2006, the UK clinical trials directive amended the Medicines and Healthcare Products Regulatory Agency (MHRA) clinical trials guidelines regarding consent of incapacitated adults without an available NOK^15^. This now permits their enrolment in trials for urgent treatments, provided there is relevant ethics committee approval. The Dex-CSDH pilot trial has this approval in the form of “Independent Healthcare Provider (IHP) consent”.
3. Finally, older patients are under-represented in medical research, even for pathologies or medicines most relevant to their age group^16–18^. As CSDH occurs almost exclusively in older patients, we aimed to demonstrate that the barriers to trial recruitment and follow-up can be overcome. Members from a local public involvement research group (INsPIRE) were involved in the trial design to help ensure it would be acceptable for our target patient group.

### Secondary pilot trial objectives

Our secondary objectives were to assess i) the feasibility of outcome measures ii) follow-up rates and iii) any early safety concerns.

Primary outcome data must be accurately and reliably collected to avoid bias from missing data. To optimise follow-up, postal and telephone questionnaires and involvement of the NOK were utilised.

An increased mortality in patients treated with steroids in the “Corticosteroid Randomisation After Significant Head injury” (CRASH) trial and the reported complication rate associated with steroid use in older patients, particularly those with co-morbidities, mandated a careful and thorough assessment of drug tolerability in this trial^19–21^. Conversely, successful and safe steroid use in older patients for treatment of polymyalgia rheumatica, temporal arteritis, chronic obstructive pulmonary disease and glioblastoma offers reassurance of beneficence^22–25^. Steroid side effects are generally dose and duration dependent^23,26^. Regimen optimisation is therefore important to minimise serious adverse events (SAEs) and maximise therapeutic benefit.

The internal pilot trial was designed using methodology concordant to the planned substantive trial, with the potential to make amendments if supported by the secondary objectives.

## Methods

The Dex-CSDH pilot trial is a randomised, double-blind, placebo-controlled trial of a two-week course of dexamethasone for 100 adult patients with a symptomatic chronic subdural haematoma (see full protocol in supplementary information). Patients were randomly assigned by an interactive web-based system to the intervention (dexamethasone) or control group (placebo). A 1:1 allocation as per a computer-generated randomisation schedule stratified by site using permuted blocks of random sizes is employed.

### Recruitment

In this pilot trial, we planned to recruit patients from up to 10 NSUs ranging in catchment population, research experience and resources, to reflect a realistic picture of multi-centre recruitment^11^. All NSUs had support from a local neurosurgical trainee acting as a co-principal investigator as part of the British Neurosurgical Trainee Collaborative (BNTRC)^27^. Hospital episode statistics (HES) indicate that a medium sized NSU admits 60–80 CSDH patients per year. Setting a conservative estimate, we predicted a recruitment rate of two patients per month for each NSU. We set an overall target of 100 patients to be recruited to the pilot within 12 months.

Eligibility criteria and overall trial design were planned in a way that would maximise participation in order to support eventual translation of findings to as broad a population as possible (Tables 1 and 2). Informed consent was obtained from the patient, NOK or IHP by an appropriately trained doctor or nurse identified on the delegation log.

**Table 1:** Dex-CSDH pilot trial inclusion and exclusion criteria.

Inclusion criteria	Exclusion criteria
Adult patients (aged 18 and older)	Patients who are already on steroids or with conditions where steroids are clearly contra-indicated
Symptomatic CSDH confirmed on cranial imaging (predominantly hypodense or isodense crescentic collection along the cerebral convexity, confirmed on CT).	Time interval from time to admission to NSU to first dose of trial medication exceeds 72 hours
Informed consent or IHP authorisation	Previous enrolment in this trial for a prior episode or concurrent enrolment in any other trial of an IMP
	Patients with CSF shunt or history of psychotic disorders
	Severe lactose intolerance or known hypersensitivity to dexamethasone or other IMP excipients, or desire to avoid gelatin

(CSDH = Chronic Subdural Haematoma, CSF = Cerebrospinal Fluid, CT = Computerised Tomography, IMP = Investigational Medicinal Product, NSU = Neurosurgical Unit, IHP = Independent Healthcare Provider).

**Table 2:** Trial design aspects to maximise broad recruitment.

Aid site engagement with recruitment	Aid recruitment of eligible patients
**Trainee Co-PI** at all sites; to support PI with site set-up administration and encourage local recruitment	**72-hour recruitment window**; from admission to NSU, allowing sufficient time to contact relatives and avoid missing patients admitted at the weekend.
**Face-to-face initiation**; to engage maximum number of people in clinical team and answer questions/concerns before starting the trial	**NOK and IHP consent**; to allow inclusion of patients lacking capacity (which would be a large proportion with this condition) and those without a NOK available
**Monthly screening logs**; to monitor screening and allow early identification of any institutional reasons for screen failures	**Medication diary**; simple diary with dates and pictures to tick off each day, reminding patients what to take (as drug regime complex and elderly patients often already have polypharmacy)

(IHP = Independent Healthcare Provider, NOK = Next-of-kin, NSU = Neurosurgical Unit, PI = Principal Investigator).

### Primary outcome measure

**Figure 2:**
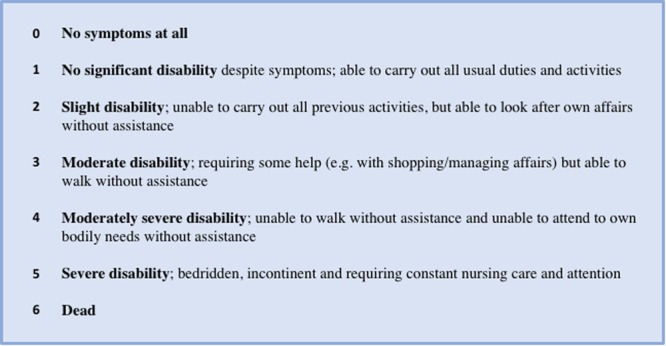
modified Rankin Scale^28^. Category 6 added to allow for mortality outcomes.

The primary outcome measure was determined as the modified Rankin Scale (mRS) at six months’ post-randomisation, as it is a core instrument for measuring the degree of disability or dependence in daily activities of living (Fig. 2)^28^. It is an ordinal scale but dichotamised values of 0–3 (good outcome) and 4–6 (poor outcome) have been used in previous CSDH research^29,30^. The final time-point of six months was selected so that most CSDH recurrences had occurred and been treated, and to permit adequate time for recovery and adaptation to disability^31^. The mRS is assessed by blinded research staff via telephone interviews or by the patient completing a structured postal questionnaire^32^.

### Secondary outcome measures

Secondary outcome measures are listed in Table 3. Amongst others, these included endpoints commonly used in the field of CSDH, such as re-operation, mortality and complications^33^.

**Table 3:** Trial outcome measures and changes made following completion of pilot.

Outcome measures (protocol version 1.0)	Changes following pilot trial
**Primary outcome measure; mRS at 6 months**	**Re-formatted**, **mRS adjudication and return window added** (**−4/+8 weeks**)
**Secondary outcome measures;**	**Return windows added: 3 months** (**+/−8 weeks) 6 months** (**−4/+8 weeks**)
No. of CSDH-related surgical interventions undertaken during the index admission and subsequent admissions in follow-up period	Unchanged
GCS at discharge from NSU and at 6 months	Unchanged
**mRS at discharge from NSU**	**Re-formatted and mRS adjudication added**
**mRS at 3 months**	**Re-formatted and mRS adjudication added**
Barthel Index at discharge from NSU, 3 months and 6 months	Unchanged
**MoCA at discharge and clinical follow-up**	**Removed**
EQ-5D at discharge, 3 months and 6 months	Unchanged
Length of stay in NSU and secondary care	Unchanged
Discharge destination from NSU	Unchanged
30-day and 6-month mortality	Unchanged
**Related complications**	**Changed AESI/SAE collection process**
Health economic analysis	Unchanged
**Exploratory outcome measures**	**Added**

(AESI = Adverse Event of Special Interest, CSDH = Chronic Subdural Haematoma, Eq. 5D = EuroQol-5D, GCS = Glasgow Coma Scale, MoCA = Montreal Cognitive Assessment, mRS = modified Rankin Scale, NSU = Neurosurgical unit, SAE = Severe Adverse Event).

### Drug regimen and safety

The Dex-CSDH trial regimen was designed by incorporating a literature review (see Table 4) with the clinical expertise of the protocol development team including neurosurgeons, elderly care specialists and pharmacists. The regimen starts with a high dose (16 mg/day) and tapers down quickly to stop over 14 days, providing an average weekly dose of 62 mg dexamethasone. This is comparable to the average steroid doses reported in previous CSDH studies, and is at the lower end of course duration, to minimise complications from prolonged use. The drug is over-encapsulated so that dexamethasone is indistinguishable from placebo.

**Table 4:** Review of dexamethasone dosing schedules, adverse events and outcomes in the CSDH literature compared to Dex-CSDH pilot trial.

Paper (year)	Patient number (follow-up)	Dex dosing schedule (average weekly dose)	Adverse events	Outcome
*Dex-CSDH pilot trial*	*100 dex and placebo* *(6 M)*	*8 mg BD for 3D*, *6 mg BD for 3D*, *4 mg BD for 3D*, *2 mg BD for 3D*, *2 mg OD for 2D (****62*** ***mg****)*	*See* Table 8 *for pilot adverse events*	*See* Fig. 5 *for pilot outcomes*.
Bender (1974)^8^	37 (mean 2.5 years)	60–120 mg prednisolone for average of 21 days (**Equivalent 70–140** **mg**)	None reported.	Reduced bed rest & hospitalisation. 71% patients avoided surgery.
Sun (2005)^3^	26 dex 69 dex & surgery 13 surgery	Dex alone: 16 mg daily for approx. 21 days. (**112** **mg**)	2/4 DM patients needed additional insulin, resolved on stopping treatment.	84% favourable outcome in dex alone. Recurrence 4% with dex versus 15% without.
Delgado-Lopez (2009)^9^	101 (median 6 M)	12 mg daily, tapering by 1 mg every 3 days (**46**.**8** **mg**)	Hyperglycaemia (14.8%) and infections (9%), 1 gastric ulcer (<1%).	78.2% dex patients avoided surgery. 96% favourable outcome with dex.
Berghauser Pont (2012)^4^	496 (3 M)	dex 16 mg daily starting median of 4 days pre-op and then weaning (**unspecified**).	Empyema 2.8% DVT/PE 1.8% Hyperglycaemia only whilst on dex.	Longer pre-operative dex dose associated with lower recurrence and no increased morbidity.
Berghauser Pont (2012)^5^	5 studies with **total 520**	Study 1–3 as per Bender, Delgado-Lopez, Sun^2,7,8^ Study 4: 16 mg/day tapering over 8wks. Study 5: 0.5 mg/kg pred = 6 mg dex/day for 4 wks.	Infections 9% GI bleed <1% (2/520) Hyperglycaemia 7.7–14.8% (higher with long-term use)	Good outcome in 83–100% with steroids and 64–92% surgery alone **Recurrence:** 4–27.8% with steroids and 15–26.3% surgery alone.
Emich (2014)^52^	820 dex and placebo (24 weeks)	6 day course of dex from 16 mg/day to, 4 mg/day. (**68** **mg**)	Trial on-going since 2014: no safety issues reported	Primary end-point will be re-operation within 12 weeks.
Chan (2015)^53^	122 dex & surgery 126 surgery alone	16 mg for 4D, 6 mg for 3D, 2 mg for 3D (**61**.**6** **mg**).	No increase in adverse events with dex	6.6% recurrence dex & surgery, 13.5% surgery only. 83–85% favourable outcome in both groups.
Thotakura (2015)^7^	26 (mean 16.5 M)	12 mg/day for 3D, then tapered over 4 weeks (**27**.**5** **mg**)	1 hyperglycaemia 1 gastritis.	42% avoided surgery
Prudhomme (2016)^54^	20 dex or placebo (6 M)	12 mg/day for 21D,tapered over 7D (**70**.**25** **mg**)	4 hyperglycaemia, 5 other SAEs	Trial halted due to high SAE rate.
Qian (2017)^6^	75 dex 167 no dex	4.5 mg TDS for 4D, weaned every 4D (**155**.**13** **mg**)	5/13 DM patients with hyperglycaemia.	Recurrence 8% with dex, 19.8% without dex.

(BD = twice a day, D = day, dex = dexamethasone, DM = Diabetes Mellitus, GI = Gastrointestinal, M = Month, OD = Once a Day, SAE = Serious Adverse Event, TDS = Three times a Day).

The pilot trial could potentially identify early safety concerns, therefore there was close monitoring of SAEs and adverse events of special interest (AESIs). The latter were adverse events we expected in relation to steroid use from our clinical experience in neurosurgery and included; hyperglycaemia requiring treatment or stopping of trial medication, new-onset diabetes, psychosis and gastric symptoms (e.g. dyspepsia, gastric ulcer).

### Progression from internal pilot to substantive trial

**Figure 3:**
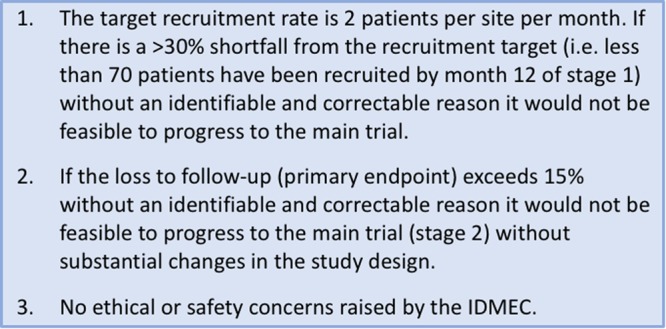
formal progression criteria for Dex-CSDH pilot trial.

Progression criteria were determined prior to initiation of the pilot (Fig. 3). These were reviewed alongside the blinded pilot trial data by the trial steering committee (TSC), and the unblinded data by the independent data monitoring and ethics committee (IDMEC). Review of blinded pilot trial data was used to enable minor protocol modifications to assist conduct of the substantive trial (see results).

### Sample size calculation and statistical analysis for the substantive trial

The overall sample size of the substantive Dex-CSDH trial is 750 patients. An 8% increase in the rate of favourable outcome (mRS 0–3) at 6 months was considered a clinically important treatment effect. On the basis of the available literature, we estimated that 80–85% of the control group will have a favourable outcome^3,29^. Using a 2-sided test at the 5% significance level, a sample of 750 patients (allowing 15% loss to follow-up) will detect an absolute difference of 8% with a power of 81–92%. The internal pilot data will be included in the substantive trial results, on an intention-to-treat analysis. Secondary endpoints will also be summarised and an economic analysis performed (see protocol for full details).

## Results

### Primary objective: Recruitment

**Figure 4:**
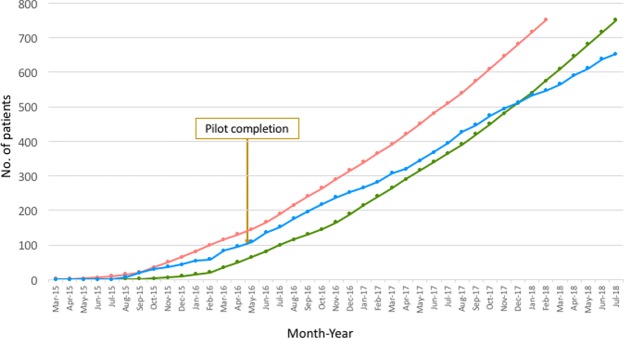
Recruitment curve. Orange line = original planned recruitment curve, Green line = same original recruitment curve pushed back 5 moths due to delay start. Blue line = actual recruitment.

Pilot trial recruitment commenced in August 2015, five months later than the original anticipated start date of March 2015 (Fig. 4). A further six sites opened to recruitment over the subsequent eight months. The 100^th^ patient was recruited within nine months of the August start date, easily surpassing the recruitment target rate and allowing progression onto the substantive trial which has now recruited 653 patients to July 2018 (Table 5).

**Table 5:** Site opening timetable and recruitment in order of site openings. *top 5 recruiting sites from substantive trial.

Site	Date of local R + D Application	Months from R + D to site opening	Pilot: All pts (n)	Pilot: mean pts/month (n)	ST: All pts to 19/7/18 (n)	ST: mean pts/month (n)
Cambridge (Lead site)	Jan 2014	19	75	**8**	243	**6**.75^*^
Plymouth	Mar 2015	7	5	0.7	32	0.9
Imperial	Aug 2015	5	2	0.5	5	0.2
Southampton	Sept 2015	4	13	**3**.**3**	74	**2**.4^*^
Middlesbrough	Oct 2015	5	2	1	21	0.7
Sheffield	Oct 2015	5	2	1	47	**1**.6^*^
Birmingham	Aug 2015	8	1	1	23	0.8
Brighton	Oct 2015	6	N/A	N/A	21	0.8
Leeds	Oct 2015	7	N/A	N/A	38	1.4
Glasgow	Oct 2015	7	N/A	N/A	52	**1**.9^*^
Stoke	Sept 2015	9	N/A	N/A	20	0.8
Preston	Sept 2015	11	N/A	N/A	7	0.3
Aberdeen	Nov 2015	9	N/A	N/A	9	0.4
Edinburgh	Nov 2015	11	N/A	N/A	11	0.5
Newcastle	Nov 2015	12	N/A	N/A	6	0.3
Dundee	Mar 2016	8	N/A	N/A	4	0.2
Hull	Dec 2015	15	N/A	N/A	12	0.7
Romford	Aug 2016	9	N/A	N/A	6	0.4
Cardiff	Sept 2017	11	N/A	N/A	1	0.1
RLH	Mar 2017	6	N/A	N/A	11	1
SGH	Feb 2016	26	N/A	N/A	7	**1**.75^*^
Oxford	Oct 2017	8	N/A	N/A	3	1.5
**Total (per site)**		**189 exc**. **lead (9 per site)**	**100**	**15**.**5 (2**.**2 per site)**	**594**	**25**.**4 (1**.**2 per site)**

(exc. = excluding, pts = patients, R + D = Research and development, ST = substantive trial).

The time from research and development (R + D) first contact to site opening was an average of 5.7 months in the pilot trial (excluding the sponsor site which required a more rigorous opening procedure), and has increased to nine months for the substantive trial (see Table 5).

Anonymised patient screening logs were available from all pilot sites; 47% of patients screened were enrolled (Table 6). A reason for screening failure was provided in 92 out of the 114 pilot patients screened (81%). The most common reason was patient or NOK declining consent (42%), followed by patient co-morbidities (19%), current steroid use (13%), outside 72 hr recruitment window (9%), NOK unable to attend for consent (8%) and other reasons (9%).

**Table 6:** Screening and recruitment rates at pilot sites.

	All Patients screened (n)	Average screened per month (n)	Patients recruited (n)	Recruitment rate (%)
Cambridge	126	14	75	60%
Plymouth	25	4	5	20%
Imperial	9	2	2	22%
Southampton	43	11	13	30%
Middlesbrough	5	2.5	2	40%
Sheffield	2	2	2	100%
Birmingham	4	4	1	25%
**Total:**	**214 (6/site)**		**100**	**47%**

### Secondary objectives: outcome measures, follow-up and safety

The outcome measures were completed centrally via postal or telephone questionnaire with few errors. The mRS questionnaire created several queries and the Montreal Cognitive Assessment (MoCA) was poorly completed therefore these two parts were reviewed for amendment (see changes to substantive trial protocol below). The remaining outcome measures were considered appropriate to the trial population and easy to collect.

On completion of the pilot, the first 31 patients had completed their primary outcome at six months and 55 patients had met the secondary 3-month outcome time point (Table 7). Retention in the pilot trial was excellent with primary outcome data received from 100% of the 30 eligible patients (one patient withdrawn). In 5% of patients, data was missing at the secondary time-point of 3-months. At six months, nearly all patients were back at home, or else the General Practitioners (GP) was aware of the patient location, enabling improved follow-up at this later time point.

**Table 7:** Results of data collection during pilot.

	To end of pilot recruitment (09-05-16)		To end of pilot follow-up period (09-11-16)	
	3-month outcome	6-month outcome	3-month outcome	6-month outcome
Patients: n	55	31	165	100
Data received: n (%)	51 (93%)	30 (97%)	143 (87%)	98 (98%)
Withdrawn: n (%)	1 (2%)	1 (3%)	4 (2%)	2 (2%)
TM or LTFU: n (%)	3 (5%)	0	18 (11%)	0

(LFTU = Lost To Follow-Up, TM = transiently missing; applicable to patients who miss 3-month outcome).

**Figure 5:**
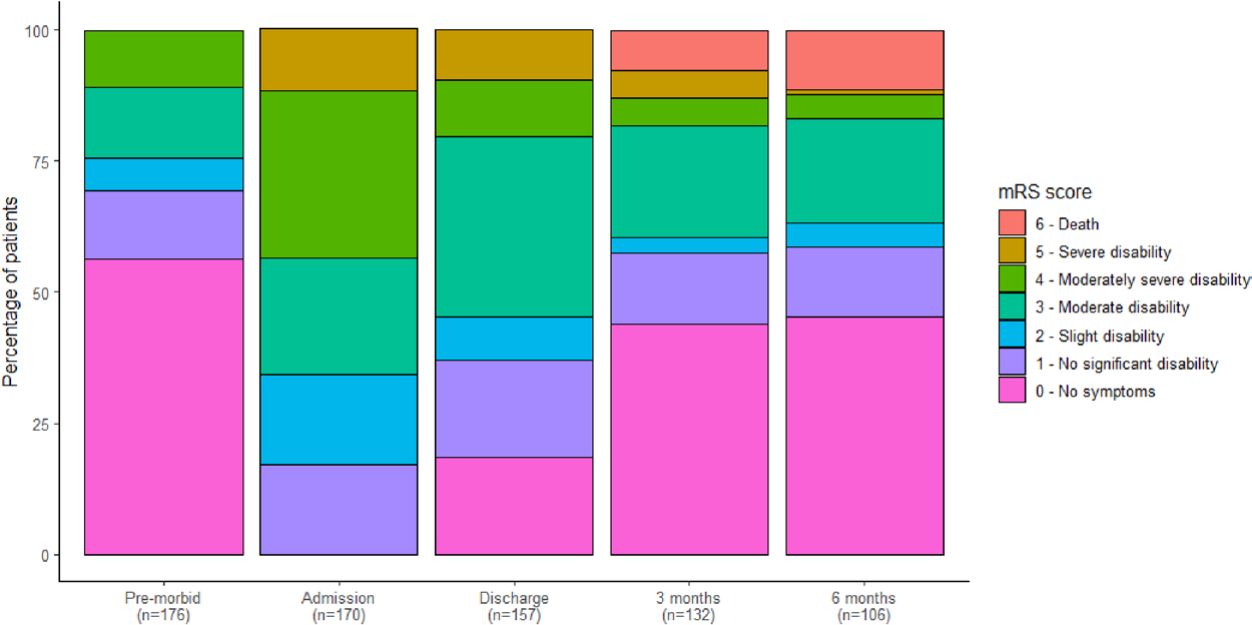
blinded mRS scores from pre-morbid state to final 6-month outcome for all data entered on pilot patients at interim analysis on 5^th^ Dec 2016. X-axis; outcome time-point, Y-axis; percentage of patients. The colours represent mRS scores as per the key.

The outcome data was reviewed again when all 100 pilot patients had met their primary end-point at 6-months and was still collected for 100% of eligible patients (two patients withdrawn). The patterns of change in mRS from data reviewed at the steering committe meeting following completion of the pilot  are summarised in Fig. 5. Collection rates of the secondary time-point of 3-months was slightly worse, with 11% of data missing.

An SAE was experienced in 10 (10%) of pilot trial patients, with one patient experiencing two SAEs (Table 8). Twelve patients (12%) experienced an AESI, with hyperglycaemia the most common event (Table 8). As only the IDMEC has access to unblinded data, it is not possible to currently state whether these AESIs occurred in patients receiving dexamethasone or placebo.

**Table 8:** Serious Adverse Events (SAEs) and Adverse Events of Special Interest (AESIs) reported during pilot.

	SAEs in pilot patients	AESI’s in pilot patients
Total no. of events:	11 events in 10 patients	12 events in 12 patients
Total no. of patients:	10/100	12/100
Event Terms:	2 Scalp lacerations	7 Hyperglycaemia requiring treatment or stopping of trial medication
	2 Acute subdural haematomas	3 Gastric reflux
	1 General physical health deterioration	1 New onset diabetes
	1 Deep Vein Thrombosis	1 Hallucinations
	1 Aspiration bronchopneumonia	
	1 Urinary Tract Infection	
	1 Fracture left Hip	
	1 Stroke	
	1 Bowel perforation secondary to Diverticulitis	

Overall, there was a 5% (5/100) withdrawal rate from the trial medication. Two patients withdrew because of hyperglycaemia. One patient withdrew following a stroke, after taking the trial medication for only one day. These three patients remained in the trial with regards to follow-up. Two further patients withdrew completely from the trial and all follow-up; one felt the tablets were too large and the other reported hallucinations after taking one dose (reported as AESI). Unblinded data was reviewed by the IDMEC and the compliance and safety profile of the trial medication was considered acceptable.

The TSC reviewed all pilot data and confirmed that as patient recruitment was feasible and there were no ethical or safety concerns, the substantive trial should take place. Recommendations were made for minor protocol amendments as detailed below. The National Institute for Health Research (NIHR) health technology assessment (HTA) programme subsequently approve progression into the substantive trial.

### Changes to substantive trial protocol based upon the pilot

#### Eligibility changes

One major change occurred to the exclusion criteria following completion of the pilot trial; from “patients who are already on steroids” to “patients who are on (or within one month of) regular oral or IV steroids”. The term “regular” was added because we noted that in some centres patients are given one dose of intra-operative dexamethasone for anti-emesis. This long-standing practice involves a one-off dose too small to have any significant impact on outcome. Nevertheless, it was agreed to collect all data on single-use steroids as a concomitant medication. The route “oral or IV” was added to clarify that patients on inhaled steroids for conditions such as asthma could be included, and a 1-month washout period of recent steroid use was also stated.

#### Outcome measures and follow-up

The pilot trial identified that remote collection of the mRS led to several data errors due to incorrect completion of this part of the questionnaire by patients or blinded assessors. The complex order of instructions led to essential questions being missed, meaning the final mRS could not be calculated without repeat patient assessment. To rectify this, the questionnaire and instructions were simplified and an adjudication process implemented where all responses were immediately reviewed by a blinded clinician in the study team, to ensure timely calculation of the mRS. Set dates for completion of questionnaires were also amended to “windows” of acceptability to minimise protocol violations and allow patients a wide opportunity to respond (Table 3).

The increase in missing data at three months (Table 7) was reviewed and identified to relate to the follow-up process performed by a central trial administrator. At the sponsor site only, the local research nurse (RN) performed follow-up and had better follow-up rates due to more timely posting of questionnaires and regular follow-up phone calls to the patient, NOK and/or GP where necessary. Therefore, it was determined that follow-up could be maximised if the sponsor site RN performed follow-up for all sites.

#### Changes to secondary outcome measures

Review of the secondary outcomes showed a paucity of data from discharge and follow-up MoCA scores . As this assessment must be done face-to-face, it was missed if patients were discharged over the weekend or with little notice. It could also not be performed during remote follow-up and very few patients attended clinic. Therefore, it was deemed that the most pragmatic solution was to remove the MoCA from the substantive trial (Table 3).

#### Changes to safety processes

As a result of three inadvertent administrations of ward stock dexamethasone rather than blinded trial medication, patient trial bracelets were introduced for the substantive trial. These clearly state that the patient is prescribed a trial medication and must not receive ward stock dexamethasone. As identity wristbands must be checked before giving any medication we expected these to deter inadvertent ward dexamethasone use.

Initially all SAEs were collected throughout the 6-month follow-up period, however all SAEs occurring more than 30 days after randomisation were considered to be unrelated to the study medication and more often related to further falls, which are common in this patient group. Limiting SAE collection to the first 30 days is in-keeping with other CSDH studies and avoids unnecessary additional patient contact, given that the 3-month and 6-month follow-up is done remotely^3^.

#### Addition of exploratory outcome measures

The original outcome measures were all clinically relevant, but none addressed the mechanistic actions of dexamethasone. The TSC agreed that exploratory outcome measures would be useful to aid scientific understanding of how dexamethasone works in CSDH, supporting its clinical application and helping direct future studies on alternative CSDH pharmacotherapies.

The first exploratory objective was to assess the biological action of dexamethasone by analysing intra-operative and post-operative blood and CSDH fluid samples; excluding patients with active malignancy or immunosuppressive therapy. A range of inflammatory markers involved in CSDH pathophysiology have already been identified^34–37^. We planned to measure a panel of these markers, assessing their response to dexamethasone exposure in trial patients and whether this related to the recurrence rate.

Dexamethasone is also well known to reduce cerebral oedema, a feature which has not previously been investigated in CSDH^38,39^. Cerebral oedema can occur due to fluid leaking through the blood-brain-barrier and has been linked to blood flow patterns in the brain (cerebral perfusion)^40–42^. There is some evidence that cerebral perfusion is globally reduced in CSDH and improves following surgical treatment^43–46^. Therefore, the second exploratory objective was to assess the role of dexamethasone in cerebral perfusion and oedema, utilising transcranial Doppler and magnetic resonance imaging (excluding patients with renal dysfunction or a pacemaker/metal implant).

## Discussion

Multicentre trials often do not meet their original target recruitment in time and must be extended^47^. In fact, our average recruitment of 2.2 patients/month per site during the pilot trial exceeded the target of 2 patients/month per site. The target was far exceeded in two of the sites (8/month in Cambridge and 3.3/month in Southampton), and was below the target in the remaining five sites. Of these five sites, two are small centres (Plymouth and Imperial) with limited populations to recruit from and the remaining three had only been open two months. Overall, we considered that our target recruitment plan from the pilot could be applied to the substantive trial, but that we would attempt to open larger centres first and implement techniques to promote recruitment at the lowest recruiting sites. Three of the seven pilot sites have gone on to be in the top five recruiting sites in terms of monthly recruitment rate for the whole trial (Table 5). This may mean that we were successful in identifying strong sites to open during the pilot period, or that the longer sites are open the better they are at recruiting.

Recruitment patterns from individual centres should be carefully observed when assessing recruitment feasibility with a pilot trial. As despite recruitment curves traditionally being exponential in design, this often does not reflect the realities of trial recruitment, which after an initial take-off can remain constant. Recruitment fatigue can also mean that previously well-recruiting centres may decline over the course of the years it takes to complete a large trial. Indeed, many of the sites we recruited after the pilot phase are recruiting less well than those opened during the pilot phase and recruiting rates in the top centres have remained stable or declined (Table 5). Overall the recruitment has declined from an average of 2.2 patients per site per month to 1.2, resulting in recruitment falling behind target in the substantive trial despite exceeding the pilot target (Fig. 4).

An average R + D set-up time of 5.7 months during the pilot meant that most sites opened later than anticipated and only two sites were open for the first six months of the pilot (Table 5). This led to a bias in the pilot recruitment with 75% of the patients recruited from the lead site (Cambridge) and only 25% from other sites. This could affect the generalisability of the trial. To address this, recruitment is continually encouraged at other sites and the TSC specified that the final trial should not have more than 50% of the patients recruited from a single site. Currently 37% of patients have been recruited from the lead site.

The top two recruiting pilot sites were also the sites screening the largest number of patients (11–14 per site per month), whilst other sites only screened 2–4 patients per month. This may relate to the staffing at these sites, as both have a research fellow and nurse dedicated to trials. This enables them to invest more time and effort in identifying, approaching and discussing the trial with potential patients. Most other centres are reliant on the clinical staff to screen and enrol patients, adding to their daily workload and therefore requiring significantly more motivation. It is also highly dependent on the availability and support from the local RN, who may not have a neuroscience affiliation. It is self-evident that limited infrastructure and research staffing will have an impact on the delivery of RCTs.

A recent review on strategies to improve recruitment to RCTs suggested that only open-label studies and telephone reminders have been shown to increase recruitment^47^. As neither of these strategies were appropriate for this blinded trial in an acute neurosurgical population, we considered that efforts would be best directed towards promoting site engagement and incentivising investigators at each site to screen and enrol patients. This included setting up a trial website; regular newsletters to all sites highlighting trials news with local and national recruitment rates; trial posters for use in clinical areas to remind clinicians about the trial; additional site visits for training; promotion of the trial at national meetings and finally an annual investigators day. Feedback from this latter initiative has been very positive and is perceived to significantly impact on site engagement.

Screening failures were reviewed to assess recruitment and any potential bias in patient selection. The most common reason for screening failure was patient or NOK declining. Aside from ensuring local teams are well trained in giving correct and detailed trial information, this is an acceptable and expected reason for screening failure. The second most common reason was patient co-morbidity and it became apparent from discussion with sites that there was variation in the assessment of this. For example, some sites were reporting general “frailty” as a common cause of exclusion, whilst other sites would be more inclusive of patients with wide ranging co-morbidities and medications. Clinical judgement is clearly required during the screening process but this does also mean that the clinicians own bias will be introduced. Indeed, it is recognised that elderly patients can be perceived by clinicians as being vulnerable and needing “protection from researchers”, despite their desire to engage in trials^17^. We specifically tried to be as pragmatic and inclusive as possible when designing the eligibility criteria, so that the trial results will be widely applicable to the elderly population affected by CSDH. We have found regular dissemination of the trial progress and low adverse event rate has helped encourage this.

In 8% of screening failures the patient was deemed to lack capacity and there was no available NOK. Patients lacking capacity are usually those with more severe CSDHs resulting in cognitive deficits. To avoid skewing the recruitment towards the CSDH patients with milder symptoms, IHP consent can be used when patients lack capacity. The pilot highlighted the need to promote this and train sites about appropriate use of IHP consent. There is evidence from a public opinion survey on patient inclusion in severe traumatic brain injury (TBI) clinical trials that 91% of respondents would be happy for an independent doctor to assent^48^. Despite this, we have found there is persistent institutional reluctance to use such proxy consent processes, which is only likely to be overcome by continued adoption into trials where appropriate.

The reliability of trial outcome measure collection was monitored during the pilot leading to a minor amendment to the mRS questionnaire, which had been incomplete in some cases. Primary outcome data was collected in 97% of patients at the end of pilot recruitment period and was maintained at 98% once all pilot patients had reached six months, with only two patients withdrawn. The internal pilot has provided a realistic estimate of retention and follow-up which has been maintained into the substantive trial. It also reflects effective trial design with regard to strategies to maintain patients in the trial. This includes the techniques discussed in Table 2, particularly ensuring that follow-up was remote, as elderly patients are more likely to participate if follow-up is done from home^49^. A review of strategies to improve retention in randomised trials also showed that financial incentives improve return of postal and electronic questionnaires, however this was not considered appropriate in an NHS setting^50^. While postal reminders have been reported to have no significant effect on follow-up, we found telephone follow-up reminders very helpful^50^. This may be specific to elderly patients who will have a higher rate of cognitive and physical impairments that make filling in and returning a written questionnaire difficult. Some patients reported preferring to answer the questionnaires over the phone.

The follow-up rate has remained excellent into the substantive trial, despite the number of trial sites increasing to 21 and extending from England into Scotland and Wales. The follow-up is now undertaken by the sponsor site RN, who dedicates a lot of time to liaising with local hospitals, GPs, patients and their NOKs to get the outcome data. A 6-month lost-to-follow-up (LTFU) rate of 0% in the pilot is exceptional and can only hope to be maintained throughout the substantive trial, however less than 5% LTFU is suggested to be acceptable in minimising bias to trial results^51^.

The pooled primary outcome (6-month mRS) was found to be favourable in 83% of pilot patients, which was close to that predicted for the control group for the sample size calculation (80–85%). As the aim of the pilot was not to assess efficacy or calculate sample size, no further analysis was done at this stage. An interim analysis with a sample size calculation once approximately 500 patients have reached their primary outcome is planned. This will permit us to make any sample size adjustments if we are close to seeing a significant treatment effect.

We anticipated a relatively high SAE rate in a trial on elderly patients with a surgical condition. This was 10% in the pilot and has remained at a comparable rate of 13% in the substantive trial to date. None of the SAEs in the pilot were considered related to the trial medication and all were events that might be expected in this cohort. Data was also collected on AESIs which were reported in 12% of pilot patients. Interestingly, the cumulative AESI rate in the most recent safety report from July 2018 is only 7% (including pilot and substantive trial data). This highlights the risks of collecting data in a small portion of patients such as a pilot which can have implications on subsequent trial conduct.

The IDMEC reviewed unblinded data on SAEs, AESIs and outcomes reported during the pilot, and had no safety or ethical concerns, recommending continuation onto the substantive trial with the same protocol.

## Conclusion

The Dex-CSDH pilot trial demonstrated feasible recruitment, with an excellent follow-up rate and no safety concerns. This supported transition into an on-going substantive trial with only minor trial amendments aimed at improving data collection, streamlining safety processes and promoting recruitment. These changes, alongside the addition of exploratory outcomes, add value and scope to this important clinical trial.

Pilot trials are useful to assess feasibility and guide conduct of the subsequent substantive trial. Careful analysis of both screening and recruitment patterns permits predictable estimation of multi-centre trial recruitment and sharing data from pilot experiences can help guide future pilot and substantive trial design.

## Supplementary information

CONSORT Checklist

Protocol version 3.0

## Data Availability

The data reported in this manuscript was analysed for internal reporting to the TSC and IDMEC and is available from the trial management group on reasonable request. On completion of the substantive trial, all data will be deposited in the University of Cambridge repository.
