# Supplementary material for: Dex-CSDH randomised, placebo-controlled trial of dexamethasone for chronic subdural haematoma: report of the internal pilot phase
Source: Sci Rep. 2019 Apr 10;9:5885. doi: 10.1038/s41598-019-42087-z (PMC6458174; doi:10.1038/s41598-019-42087-z)

A randomised, double blind, placebo-controlled trial of a two-week course of dexamethasone for adult patients with a symptomatic Chronic Subdural Haematoma (Dex-CSDH trial).

Dex – CSDH Trial

CLINICAL TRIAL PROTOCOL

Protocol version: 3.0

Protocol date: 27 April 2017

This document contains confidential information. This document must not be disclosed to anyone other than the trial staff and regulatory authorities. The information in this document cannot be used for any purpose other than the evaluation or conduct of the clinical investigation without the prior written consent of Professor PJ Hutchinson and the Sponsor.

Clinical Trial Protocol

Trial Title:	A randomised, double blind, placebo-controlled trial of a two-week course of dexamethasone for adult patients with a symptomatic Chronic Subdural Haematoma (Dex-CSDH trial).
Protocol Acronym:	Dex-CSDH
EudraCT Number:	2014-004948-35
ISRCTN:	80782810
Investigational Product:	Dexamethasone
Protocol Version:	3.0 dated 27 April 2017

Chief Investigator:	Professor Peter Hutchinson
CI Address:	Division of Neurosurgery, Box 167, University of Cambridge, Cambridge Biomedical Campus, Cambridge, CB2 0QQ
Telephone:	+ 44 (0)1223 336946
Fax:	+ 44 (0)1223 216926
Co-ordinating Centre:	Cambridge Clinical Trials Unit (CCTU)<br>Coton House, Level 6, Box 401, Addenbrooke's Hospital, Hills Road, Cambridge, CB2 0QQ<br>Telephone: + 44 (0)1223 254921<br>Fax: + 44 (0)1223 256763<br>Email:carol.daviswilkie@addenbrookes.nhs.uk
Trial Sponsor:	Cambridge University Hospitals NHS Foundation Trust and University of Cambridge
SAE Reporting:	Cambridge Clinical Trials Unit (CCTU)<br>Clinical Trial Coordinator (CTC): Carol Davis Wilkie<br>Telephone: + 44 (0)1223 254921<br>Fax: 01223 254247<br>Email: DEX-CSDH_Mailbox@addenbrookes.nhs.uk<br>OR carol.daviswilkie@addenbrookes.nhs.uk<br>OR carol.davis-wilkie@nhs.net

Funder:	NIHR Health Technology Assessment Programme

1 Protocol Signatures:

I give my approval for the attached protocol entitled *"A randomised, double blind, placebo-controlled trial of a two-week course of dexamethasone for adult patients with a symptomatic chronic Subdural Haematoma (Dex-CSDH trial)"* dated 27 Apr 2017.

Chief Investigator:

Name: Professor Peter Hutchinson

Signature: PJA Hutch

Date: 17.05.2017

Trial Statistician:

Name: Dr Simon Bond

Signature: [signature]

Date: 17 May 2017

Site Signature

I have read the attached protocol entitled *"A randomised, double blind, placebo-controlled trial of a two-week course of dexamethasone for adult patients with a symptomatic chronic Subdural Haematoma (Dex-CSDH trial)"* dated 27 Apr 2017 and agree to abide by all provisions set forth therein.

I agree to comply with the conditions and principles of Good Clinical Practice as outlined in the European Clinical Trials Directives 2001/20/EC and the GCP Directive 2005/28/EC.

I agree to ensure that the confidential information contained in this document will not be used for any other purpose other than the evaluation or conduct of the clinical investigation without the prior written consent of the Professor Peter Hutchinson and the Sponsor

Principal Investigator (PI):

Name: ______________________

Signature: ______________________

Date: ______________________

2 Trial Management Committee and Protocol Contributors

Chief Investigator
Professor Peter Hutchinson
Professor of Neurosurgery & Honorary Consultant Neurosurgeon
University of Cambridge & Cambridge University Hospitals NHS Foundation Trust
Division of Neurosurgery, Box 167, University of Cambridge, Cambridge Biomedical Campus, Cambridge, CB2 0QQ
Telephone: + 44 (0)1223 336946
Fax: + 44 (0)1223 216926
Email: pjah2@cam.ac.uk

Trial Statistician
Dr Simon Bond
Senior Statistician in the Cambridge Clinical Trials Unit (CCTU)
Coton House, Level 6, Box 401, Addenbrooke’s Hospital, Hills Road, Cambridge, CB2 0QQ
Telephone: + 44 (0)1223 596475
Email: simon.bond@addenbrookes.nhs.uk

Independent TSC Chair
Prof B Anthony Bell
Professor of Neurosurgery
St George's University of London
Academic Neurosurgery Unit
St George's University of London
Cranmer Terrace
London SW17 0RE
Telephone +44(0) 2087254179
Fax +44 (0) 2087255139
Email: neurosur@sgul.ac.uk

Independent DMC Chair
Prof Martin Smith
Consultant & Honorary Professor in Neurocritical Care
University College London Hospitals
Box 30, Department of Neurocritical Care, National Hospital for Neurology and Neurosurgery, Queen Square, London, WC1N 3BG
Telephone +44 (0)2078298711
Email: martin.smith@uclh.nhs.uk

Trainee Representatives
Mr Angelos Kolias
Clinical Research Associate & Neurosurgical Registrar
University of Cambridge & Cambridge University Hospitals NHS Foundation Trust
Division of Neurosurgery, Box 167, University of Cambridge, Cambridge Biomedical Campus,
Cambridge, CB2 0QQ
Telephone: + 44 (0)1223 746452
Fax: + 44 (0)1223 216926
Email: ak721@cam.ac.uk

Mr Hani Marcus (protocol contributor)
Clinical Research Fellow & Neurosurgical Registrar
Imperial College Healthcare NHS Trust
South Kensington Campus, London, SW7 2AZ
Telephone: + 44 (0)2075 895111
Email: hani.marcus10@imperial.ac.uk

Mr Paul Martin Brennan(protocol contributor)
Clinical Lecturer & Neurosurgical Registrar
Department of Clinical Neurosciences, University of Edinburgh, Western General Hospitals NHS Trust
Crewe Road, Edinburgh EH4 2XU
Telephone: + 44 (0)1317 773500
Email: paul.brennan@ed.ac.uk

Mr Aswin Chari (protocol contributor)
Division of Neurosurgery, Box 167, University of Cambridge, Cambridge Biomedical Campus,
Cambridge, CB2 0QQ
Email:aswinchari@doctors.org.uk

Mrs E Edlmann (Research Fellow)
Clinical research fellow and Neurosurgical Registrar
Department of Clinical neuroscience, Box 167, University of Cambridge, Cambridge Biomedical Campus,
Cambridge, CB2 0QQ
Telephone: + 44 (0)7834366709
Email: eedlmann@nhs.net

Neurosurgical Expertise
Professor Andrew King (protocol contributor)
Consultant Neurosurgeon
Department of Neurosurgery, Salford Royal Hospitals NHS Foundation Trust
Stott Lane, Salford, Manchester, M6 8HD
Telephone: +44 (0)1612 065541
Email: andrew.king@manchester.ac.uk

Mr Diederik Bulters (protocol contributor)
Consultant Neurosurgeon & Honorary Senior Clinical Lecturer
University Hospital Southampton NHS Foundation Trust Tremona Rd,
Southampton, Hampshire SO16 6YD
Telephone: + 44 (0)2380 777222
Email: dbulters@nhs.net

Mr Patrick Mitchell (protocol contributor)
Consultant Neurosurgeon
The Newcastle upon Tyne Hospitals NHS Foundation Trust
Royal Victoria Infirmary, Queen Victoria Road, New Victoria Wing, Royal Victoria Infirmary, Newcastle Upon Tyne, NE1 4LP
Telephone: +44 (0)191 233 6161
Email: patrick.mitchell@nuth.nhs.uk

Mr Dipankar Nandi (protocol contributor)
Consultant Neurosurgeon & Reader in Neurosurgery Imperial College London
Division of Neurosciences and Mental Health, Charing Cross Hospital Campus, Fulham Palace Road,
London W6 8RF
Telephone: + 44 (0)20 8846 1182
Email: d.nandi@imperial.ac.uk

Aging Research Expertise
Professor Carol Brayne (protocol contributor)
Professor of Public Health Medicine
Cambridge Institute of Public Health
Cambridge Biomedical Campus, Cambridge, CB2 0SR
Telephone: + 44 (0)1223 330334
Email: carol.brayne@phpc.cam.ac.uk

Dr Thais Minett (protocol contributor)
Senior Research Associate & Neurologist
University of Cambridge,
Department of Public Health and Primary Care,
Strangeways Research Laboratory,
2 Worts' Causeway, Cambridge CB1 8RN
Telephone: + 44 (0)1223 748600
Email: tsm34@medschl.cam.ac.uk

Surgical Expertise
Mr Antonio Belli (protocol contributor)
Reader & Honorary Consultant Neurosurgeon
NIHR Surgical Reconstruction and Microbiology Research Centre & University Hospitals Birmingham NHS Foundation Trust
School of Clinical and Experimental Medicine, University of Birmingham, Institute of Biomedical Research (West), Room WX 2.61, Edgbaston, Birmingham, B15 2TT
Telephone: + 44 (0)1214 144497
Fax: + 44 (0)1214 148867
Email: a.belli@bham.ac.uk

Clinical Pharmacology Expertise
Professor Ian Wilkinson (protocol contributor)
Professor of Clinical Pharmacology & Director of the Cambridge Clinical Trials Unit (CCTU)
University of Cambridge & Cambridge University Hospitals NHS Foundation Trust
Coton House, Level 6, Box 401, Cambridge, CB2 0QQ
Telephone: + 44 (0)1223 336898
Email: ibw20@cam.ac.uk

Clinical Trial Pharmacist
Dr Lynne Whitehead (protocol contributor)
Clinical Trials Pharmacist
Central Pharmacy, Box 55
Cambridge University Hospitals NHS Foundation Trust
Cambridge Biomedical Campus,
Cambridge, CB2 0QQ
Telephone: +44 (0)1223 216057
Email:
lynne.whitehead@addenbrookes.nhs.uk

Society of British Neurological Surgeons Research Development& Clinical Trials Manager
Mrs Carole Turner
University of Cambridge
Division of Neurosurgery, Box 167,
University of Cambridge, Cambridge Biomedical Campus, Cambridge, CB2 0QQ
Telephone: + 44 (0)1223 217205
Fax: + 44 (0) 1223 216926
Email: clt29@medschl.cam.ac.uk

Elderly Medicine/Stroke Expertises

Professor Phyo Kyaw Myint (protocol contributor)
Clinical Chair in Medicine of Old Age
School of Medicine, Health Policy and Practice
University Court of the University of Aberdeen
King's College, Aberdeen, AB24 3FX
Telephone: +44 (0)1224 437841
Email: phyo.myint@abdn.ac.uk

Dr Elizabeth Warburton (protocol contributor)
Consultant in Stroke Medicine
Department of Clinical Neurosciences
Cambridge University Hospitals NHS Foundation Trust
University of Cambridge, Cambridge Biomedical Campus, Cambridge, CB2 0QQ
Telephone: +44 (0)1223 217091
Email: eaw23@medschl.cam.ac.uk

Service User Representatives

Mr Andrew Gardner (protocol contributor)
Chief Executive Officer
Age UK Essex
112 Springfield Road, Chelmsford, Essex, CM2 6LF
Telephone: +44 (0)1245 346 106
Email: Andrew.gardner@ageukessex.org.uk

Mrs Kate Massey (protocol contributor)
Server User Representative
INsPIRE Public Involvement in Research Group for Cambridgeshire and Bedfordshire
Email: kate.massey1@btinternet.com

Health Economics Expertise

Dr Garry Barton
Reader in Health Economics
University of East Anglia
Norwich Medical School, School 2.37
University of East Anglia, Norwich, NR4 7TJ
Telephone: + 44 (0)1603 591936
Email: g.barton@uea.ac.uk

Table of Contents

1 Protocol Signatures: ... 3
Table of Contents ... 7
3 Abbreviations ... 9
4 Trial Synopsis ... 11
5 Trial Flow Chart: Dex-CSDH trial CONSORT Diagram ... 15
6 Introduction ... 16
6.1 Background ... 16
6.2 Data from non-clinical studies ... 17
6.3 Clinical Data ... 18
7 Rationale for Trial ... 19
8 Trial Design ... 20
8.1 Statement of design ... 20
8.2 Number of Centres ... 20
8.3 Number of Patients ... 20
8.4 Patients Trial duration ... 20
8.5 Trial objectives ... 20
8.6 Trial Outcome Measures ... 21
9 Selection and withdrawal of patients ... 22
9.1 Inclusion Criteria ... 22
9.2 Exclusion Criteria ... 22
9.3 Treatment Assignment and Randomisation Number ... 23
9.4 Method of Blinding: ... 23
9.5 Emergency Unblinding ... 24
9.6 Patient withdrawal criteria ... 24
10 Trial Treatments: ... 25
10.1 Dosage schedules ... 25
10.2 Presentation of the drug ... 26
10.3 Known drug reactions & interaction with other therapies ... 26
10.4 Dosage modifications ... 27
10.5 Legal status of the drug ... 27
10.6 Drug storage and supply ... 28
10.7 Concomitant therapy ... 28
11 Procedures and assessments ... 28
11.1 Screening evaluation ... 28
11.2 Baseline assessments ... 32
11.3 Exploratory assessment procedures ... 32
11.4 Trial assessments ... 33
11.5 Schedule of Assessments ... 37
11.6 Trial restrictions ... 37
12 Assessment of Safety ... 37
12.1 Definitions ... 37
12.2 Expected Adverse Reactions/Serious Adverse Reactions (AR /SARs) 39
12.3 Expected Adverse Events/Serious Adverse Events (AE/SAE) ... 39
12.4 Evaluation of adverse events ... 40
12.5 Reporting serious adverse events (SAEs) ... 41
12.6 Suspected Unexpected Serious Adverse Reactions (SUSARs) ... 41
12.7 Pregnancy Reporting ... 43
13 Toxicity – Emergency Procedures ... 43
14 Evaluation of Results (Definitions and response/evaluation of outcome measures) ... 43
14.1 Response criteria ... 43
15 Storage and Analysis of Samples ... 43

16 Statistics 43
16.1 Statistical methods 43
16.2 Interim analyses 44
16.3 Number of Patients to be enrolled 44
16.4 Criteria for the premature termination of the trial 45
16.5 Procedure to account for missing or spurious patient data 45
16.6 Definition of the end of the trial 45
17 Data handling and record keeping 45
17.1 CRF 45
17.2 Source Data 46
17.3 Data Protection & Patient Confidentiality 46
18 Trial Steering Committee and Data Monitoring and Ethics Committee 46
19 Ethical & Regulatory considerations 46
19.1 Consent 46
19.2 Ethical committee review 47
19.3 Regulatory Compliance 47
19.4 Protocol Amendments 47
19.5 Peer Review 47
19.6 Declaration of Helsinki and Good Clinical Practice 48
19.7 GCP Training 48
20 Sponsorship, Financial and Insurance 48
21 Monitoring, Audit & Inspection 48
22 Protocol Compliance and Breaches of GCP 48
23 Publications policy 49
References 50
24 Appendices 52
24.1 Appendix 1 - Trial Management / Responsibilities 52
24.2 Appendix 2 – Authorisation of Participating Sites 53
24.3 Appendix 3 - Safety Reporting Flow Chart 55

3 Abbreviations

ACTH	Adrenocorticotropic hormone
AE	Adverse Event
AESI	Adverse event of special interest
ALP	Alkaline Phosphatase
ALT	Alanine Aminotransferase
AR	Adverse Reaction
AST	Aspartate Aminotransferase
BBB	Blood brain barrier
BD	Twice daily
Bili	Bilirubin
BP	Blood Pressure
CA	Competent Authority
CBF	Cerebral Blood Flow
CCTU	Cambridge Clinical Trials Unit
CI	Chief Investigator
CRF	Case Report Form
CRH	Corticotrophin releasing hormone
CSDH	Chronic Subdural Haematoma
CSF	Cerebrospinal fluid
CT	Computerised Tomography
CTA	Clinical Trial Authorisation
CTC	Clinical Trial Co-ordinator (Central)
CTS	Corticosteroids
Dex	Dexamethasone
(I)DMC	Independent Data Monitoring Committee
DSUR	Development Safety Update Report
EDTA	Ethylenediamine tetraacetic acid
EQ-5D	EuroQol Quality of Life questionnaire
FSR	Final Trial Report
GI	Gastrointestinal
GCS	Glasgow Coma Scale
GP	General Practitioner
GCP	Good Clinical Practice
Hb	Haemoglobin
Hct	Haematocrit
HDL	High-density lipoprotein
HES	Hospital Episode Statistics
HRA	Health Research Authority
HTA	Health Technology Assessment
HPA-axis	Hypothalamo pituitary adrenal-axis
IB	Investigator's Brochure
ICH	Intracerebral haemorrhage
IDMC	Independent Data Monitoring Committee
IHP	Independent healthcare professional
IM	Intramuscular
IMP	Investigational Medicinal Product
IMPD	Investigational Medicinal Product Dossier
INR	International Normalized Ratio

ISF	Investigator Site File
IUD	Intrauterine Device
IV	Intravenous
IWRS	Interactive Web-based Response System
LDL	Low-density lipoprotein
MHRA	Medicines and Healthcare products Regulatory Agency
MoCA	Montreal Cognitive Assessment
MRI	Magnetic Resonance Imaging
mRS	Modified Rankin Scale
NG	Nasogastric
NHS	National Health Service
NICE	National Institute for Health and Care Excellence
NIHR CCRN	National Institute of Health Research Comprehensive Clinical Research Network
NIMP	Non Investigational Medicinal Product
NOK	Next of Kin
NSAIDs	Non-steroidal Anti-inflammatory Drugs
NSU	Neurosurgical unit
OD	Once daily
OPA	Out-patient appointment
PI	Principal Investigator
Plts	Platelets
PSS	Personal Social Services
QALY	Quality-adjusted life year
QP	Qualified Person
R&D	Research and Development
RA	Regulatory Agency
REC	Research Ethics Committee
RFH	Royal Free Hospital
RSI	Reference Safety Information
RTC	Randomised Controlled Trial
SAE	Serious Adverse Event
SAR	Serious Adverse Reaction
SSF	Trial Screening Form
SmPC	Summary of Product Characteristics
SOPs	Standard Operating Procedures
SUSAR	Suspected Unexpected Serious Adverse Reaction
TCD	Transcranial Doppler
TG	Thyroglobulin
TMG	Trial Management Group
TSC	Trial Steering Committee
WBC	White Blood Cells

4 Trial Synopsis

**Title of Clinical Trial**	A randomised, double blind, placebo-controlled trial of a two-week course of dexamethasone for adult patients with a symptomatic Chronic Subdural Haematoma (Dex-CSDH trial).
**Sponsor Name**	Cambridge University Hospitals NHS Foundation Trust and the University of Cambridge.
**EudraCT Number**	2014-004948-35
**Medical Condition Under Investigation**	Chronic Subdural Haematoma (CSDH).
**Purpose of Clinical Trial**	To investigate whether dexamethasone can improve the 6 month functional outcome of patients with symptomatic CSDH by reducing the rate of surgical intervention and the recurrence rate.
**Primary Objective**	To determine the clinical effectiveness of a two-week course of dexamethasone for adult patients with a symptomatic CSDH.
**Secondary Objective (s)**	1. Compare the adverse events and complications between the two arms.<br>2. Undertake a detailed economic evaluation.
**Exploratory objectives (sub-study)**	1. Assess the biological action of dexamethasone within the CSDH.<br>2. Assess the role of dexamethasone in cerebral perfusion and oedema in CSDH.
**Trial Design**	Dex-CSDH is a multi-centre, pragmatic, parallel group, double-blind, randomised trial. It will commence with Stage 1 (feasibility trial) which will be followed by the Stage 2 (substantive trial) if the progression criteria are met.
**Trial Outcome Measures**	Primary outcome measure:<br>Modified Rankin Scale (mRS) at 6 months<br><br>Secondary outcome measures:<br>- Number of CSDH-related surgical interventions undertaken during the index admission.<br>- Number of CSDH-related surgical interventions undertaken during subsequent admissions in the follow-up period.<br>- Glasgow Coma Scale (GCS) at

	discharge from NSU and at 6 months.<br>- mRS score at discharge from NSU and at 3 months.<br>- Barthel Index at discharge from NSU, 3 months and 6 months.<br>- Mortality (30-day and 6 months).<br>- EuroQOL (EQ-5D) at discharge from NSU, 3 months and 6 months.<br>- Length of stay in NSU.<br>- Discharge destination from NSU.<br>- Length of stay in secondary care.<br>- Health-economic analysis.<br>- Adverse events<br><br>Exploratory outcome measures (sub-study only);<br>- Assessment of inflammatory mediators in CSDH fluid and blood.<br>- Cerebral perfusion measured on MRI and TCD<br>- Cerebral swelling measured on MRI
**Sample Size**	Recruitment of 750 patients in the randomised trial (100 patients in during Stage 1 and 650 patients during Stage 2).
**Summary of Eligibility Criteria**	The trial focuses on adult patients with a symptomatic CSDH confirmed on cranial imaging.<br><br>Inclusion Criteria:<br>- Adult patients (aged 18 years and older).<br>- Symptomatic CSDH confirmed on cranial imaging (e.g. CT/MRI - predominantly hypodense or isodense crescentic collection along the cerebral convexity on CT).<br>- Informed consent or IHP authorisation<br><br>Exclusion Criteria<br>- Patients with conditions where steroids are clearly contra-indicated<br>- Patients who are on (or within 1 month of) regular oral or intravenous glucocorticoid steroids.<br>- Previous enrolment in this trial for a prior episode<br>- Time interval from the time of admission to NSU to first dose of trial medication exceeds 72 hours<br>- CSDH in presence of a cerebrospinal fluid (CSF) shunt.<br>- Severe lactose intolerance or any known hypersensitivity to dexamethasone or any of the other IMP (investigational medicinal product) excipients<br>- Patients with a previous history of psychotic

	disorders<br>- Unwillingness to take products containing gelatin<br>-Concurrent enrolment in any other trial of an investigational medicinal product<br>- Active malignancy (sub-study only)<br>- Currently receiving immunosuppressive drug therapy (sub-study only)<br>- Renal dysfunction (MRI sub-study only)<br>- Pacemaker or metal implants (MRI sub-study only)
**Investigational Medicinal Product and Dosage**	Dexamethasone 2mg capsule for oral or nasogastric administration.
**Comparator Product**	Placebo capsule to match active IMP for oral or nasogastric administration.
**Route(s) of Administration**	Oral or via nasogastric tube.
**Maximum Duration of Treatment of a Patient**	14 days.
**Procedures: Screening & Enrolment**	Review of routine clinical CT scan Screening will be undertaken by a member of the clinical team. Informed consent will be obtained from the patient, patient's representative (if the patient lacks capacity) or by agreement with an independent clinician.<br>In Cambridge only patients eligible for the trial, will also be screening by a member of the research team for inclusion in sub-studies and consented appropriately.
**Baseline (if different from screening and enrolment)**	• Inclusion/exclusion criteria review<br>• Informed consent process followed and consent or authorisation for enrolment obtained.<br><br>*Standard of Care*<br>• Patient medical history (including co-morbidities and relevant medications), and patient demography<br>• Injury related events - start date of suspected/proven CSDH and/or start of symptoms or suspected injury<br>• Neurological status<br>• Imaging modality and date of examination
**Treatment Period**	14 days
**End of Trial**	6 months from randomisation
**Health Technology being Assessed**	Cost utility analysis on the effectiveness of a two-week course of dexamethasone
**Procedures for Safety**	Unblinded results will be forwarded to the Data Monitoring Committee (DMC) who will

**Monitoring During Trial**	address safety issues. Any significant adverse results will be reported to the DMC via the Trial Coordinating Centre (CCTU). Onward reporting to the TSC and Sponsor.
**Criteria for Withdrawal of Patients**	Patients will be reviewed locally and patient withdrawal will be at the discretion of the treating team and local PI.<br><br>Patients requiring nasogastric administration of IMP following transfer to another hospital will be withdrawn from IMP treatment. However they will remain in the trial and data will continue to be collected until completion.

5 Trial Flow Chart: Dex-CSDH trial CONSORT Diagram

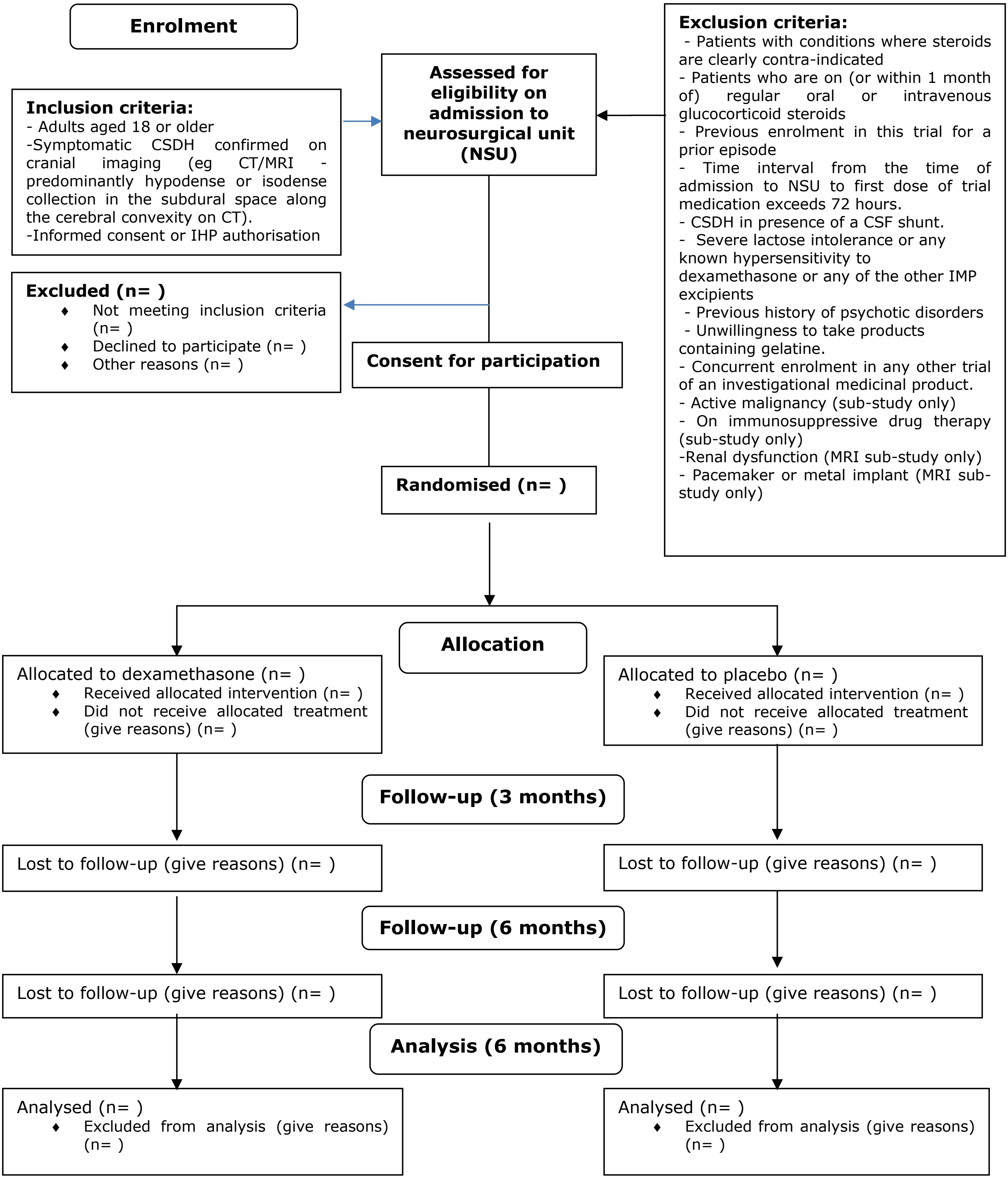

6 Introduction

6.1 Background

Chronic subdural haematoma (CSDH) is defined as a predominantly hypodense or isodense collection in the subdural space along the cerebral convexity on computerised tomography (CT). It is especially common in older patients and can happen with only a minor injury to the head even in the absence of trauma. Symptoms that can be attributed to a CSDH include headache, gait disturbance, falls, confusion/cognitive decline, focal neurological deficit, speech disturbance, drowsiness/decreased consciousness and seizures.

In the UK, 5,000 people aged over 65 years are diagnosed with a CSDH each year. In the National Health Service (NHS), patients with severe symptoms usually undergo an operation to evacuate the CSDH. Patients with milder symptoms are usually actively monitored. Although about 80% of the patients tend to recover well from this operation, up to 20% of patients will have a recurrence of the CSDH and require further operation. This significantly reduces the chances of recovery.

Dex-CSDH trial (**DEX**amethasone in **C**hronic **S**ub**D**ural **H**aematoma) is a multi-centre, clinical phase III, randomised, double blind, placebo controlled trial of dexamethasone for up to 2 weeks, on clinical outcome following CSDH. 750 patients with a clinical and radiological diagnosis of CSDH will be recruited from neurosurgical units based within the United Kingdom and Republic of Ireland. The primary outcome measure is the modified Rankin Scale (mRS) at 6 months; secondary outcome measures seek differences in the acute clinical course, mortality, length of stay in hospital, discharge destination and adverse events and complications.

Dexamethasone is a potent synthetic member of the glucocorticoid class of steroid drugs that has anti-inflammatory and immunosuppressant effects. It is 25 times more potent than cortisol in its glucocorticoid effect, while having minimal mineralocorticoid effect. Since its introduction in 1959, dexamethasone has been effectively used in a wide variety of severe and/or serious conditions for which effective treatment had not been available before the introduction of corticosteroids (CTS). If used appropriately alone or with other therapy, the benefit/risk ratio is largely positive.

The prevailing hypothesis on formation of CSDH is that following a traumatic injury, an inflammatory reaction is initiated which drives growth of abnormal blood vessels and fluid accumulation over the brain surface. This has been supported by findings of high levels of inflammatory markers within CSDH fluid [1, 2, 3, 4]. Steroids, such as dexamethasone, are thought to help turn off this inflammatory reaction, allowing better resolution of the CSDH and lower likelihood of recurrence.

Dexamethasone is also well known to reduce brain swelling, a feature which hasn't been previously investigated in CSDH. Brain swelling can occur due to fluid leaking through the membranes surrounding the brain and has also been linked to blood flow patterns in the brain (cerebral perfusion). We know cerebral perfusion is globally reduced in CSDH and improves following surgical treatment [5]. We therefore intend to investigate the relationship between cerebral oedema, how leaky the brain membranes are and cerebral perfusion on patients treated with dexamethasone versus placebo.

The trial will therefore have a significant impact on the treatment of patients as the results will determine whether steroids should be prescribed routinely for patients with symptomatic CSDH. If steroids are found to be effective, a significant impact on the

speed of recovery and functional outcome of patients is expected. Additionally, this could reduce the incidence of surgical intervention required and the length of hospital stay. As well as the impact on clinical outcome there are health economic considerations that will be addressed by the trial.

Mechanism of action
Glucocorticoid receptors are found intracellularly in almost all tissues. Glucocorticoids enter cells through passive diffusion and form a complex with a receptor protein. This complex then undergoes an irreversible activation and enters the cell nucleus, where it binds to DNA, leading to biological effects induced by these hormones, including increased hepatic gluconeogenesis, increased lipolysis, muscle catabolism, and inhibition of peripheral glucose uptake in muscle and adipose tissue. The exact mechanism of action of corticosteroids remains unknown despite more than 40 years of research.

To synthesize dexamethasone, 16β-methylprednisolone acetate is dehydrated to the 9,11-dehydro derivative. This is then reacted with a source of hypobromite, such as basic N-bromosuccinimide, to form the 9α-bromo-11β-hydrin derivative, which is then ring-closed to an epoxide. A ring-opening reaction with hydrogen fluoride in tetrahydrofurangivesdexamethasone.

Figure 1 Dexamethasone

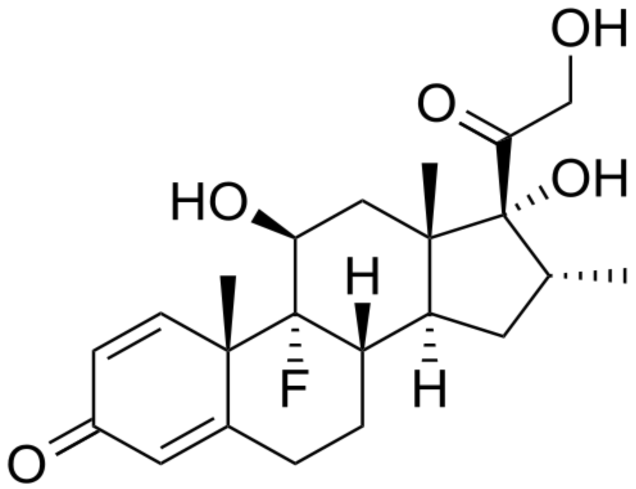

6.2 Data from non-clinical studies

Experimental animal studies on dexamethasone have revealed that it possesses greater anti-inflammatory activity than many steroids. Veterinary clinical evidence indicates dexamethasone has approximately twenty times the anti-inflammatory activity of prednisolone and 70 to 80 times that of hydrocortisone. Thymus involution studies show dexamethasone possesses 25 times the activity of prednisolone. In reference to mineralocorticoid activity, dexamethasone does not cause significant sodium or water retention. Metabolic balance studies show that animals on controlled and limited protein intake will exhibit nitrogen losses on exceedingly high dosages.

The half-life of dexamethasone 21-isonicotinate was determined in human, rat, and rabbit sera. In rat and rabbit sera 90 and 99% of the ester was hydrolyzed after 10 minutes, respectively. The half-life of in human serum was about 90-100 minutes

[6].Dexamethasone trimethylacetate was rapidly hydrolyzed to dexamethasone in bovine and equine whole blood; half-lives ranged from 10-30 minutes for both species [7]. Dexamethasone imethylbutyrate was hydrolyzed quickly in bovine plasma, with a half-life of about 1 hour [8].

6.3 Clinical Data

6.3.1 Efficacy

There is evidence from our previous trial of CSDH that the use of an intervention to reduce recurrence (subdural drain) also resulted in reduction in mortality and rate of poor functional outcome at 6 months [9]. A considerable body of evidence suggests that administration of steroids can reduce the recurrence rate and the rate of surgical interventions [10-14]. This, in turn, can reduce mortality and morbidity, and improve long-term functional outcome and quality of life of patients with CSDH. The dose and duration of dexamethasone are reflective of other recent studies in the field [15]. Moreover, a maximum of a two-week course of dexamethasone provides the best balance in terms of clinical efficacy and risks [16].

However, at present, there is no high-quality evidence to support the use of steroids in the setting of CSDH. Given the potential benefits to the patients and the potential cost savings, this is an important clinical question to address for the NHS. It is also reflected in the worldwide interest in the condition, as three clinical trials investigating the role of steroids in CSDH are registered with the WHO International Clinical Trials Registry Platform [17]; the only multi-centre trial (Austria and China) is focusing on patients undergoing an operation for CSDH [15].

6.3.2 Safety and tolerability

In general, glucocorticoid dosage depends on the severity of the condition and response of the patient. The overall benefit/risk ratio of dexamethasone continues to be beneficial.

6.3.3 Pharmacokinetics & pharmacodynamics

Dexamethasone is one of the most potent synthetic analogues of the naturally occurring glucocorticoid, hydrocortisone. It has 6-7 times the anti-inflammatory potency of prednisolone and 25 times that of hydrocortisone [16]. Dexamethasone has practically no water- and salt- retaining properties, and is therefore suitable for use in patients with cardiac failure or hypertension.

Pharmacokinetics

Absorption
Dexamethasone is rapidly and well (around 80%) absorbed when given by mouth. Peak plasma levels are reached between 1 and 2 hours after ingestion of dexamethasone tablets. After intramuscular (IM) and intravenous (IV) administration of water soluble dexamethasone sodium phosphate the onset of action is relatively fast [18].

Distribution
In general, glucocorticoids are readily absorbed from the gastro-intestinal tract. They are also well-absorbed from sites of local application. They are rapidly distributed to all body tissues. They cross the placenta and are excreted in small amounts in breast milk [18]. Dexamethasone is reversibly bound (up to 77%) to plasma proteins, mainly

globulin and less so to albumin. Only the fraction of the corticosteroids that is unbound can enter cells to mediate corticosteroid effects [19]

Metabolism
Corticosteroids are metabolised mainly in the liver but also in the kidney, and are excreted in the urine. The slower metabolism of the synthetic corticosteroids with their lower protein-binding affinity may account for their increased potency compared with the natural corticosteroids. Dexamethasone metabolism in the liver is slow and rather limited. Studies suggest that the major metabolic pathway involves the formation of un-conjugated polar metabolites.

Excretion
The plasma half-life of dexamethasone is 3-4.5 hours, but as the effects significantly outlast plasma concentrations of steroids, the plasma-half life is of little relevance and the use of the biological half-life is more applicable. The biological half-life of dexamethasone is 36-54 hours [18].

In patients with liver disease dexamethasone clearance was reduced, due to an impairment of metabolism. In renal failure, on the other hand, clearance was increased due to acceleration of metabolism [20]. The major route of excretion of dexamethasone and its metabolites is via the kidneys. After oral administration a large proportion (30%) of the total dose administered is likely to be excreted in the urine as unchanged dexamethasone.

Pharmacodynamics
In humans, cortisol is the main glucocorticoid. Glucocorticoids are produced and secreted by the adrenal cortex and are an intrinsic part of the hypothalamo pituitary adrenal-axis (HPA-axis). Fluctuations in the rates of secretion of glucocorticoids are determined by fluctuations in the release of adrenocorticotropic hormone (ACTH) by the pituitary corticotropes.

These corticotropes, in turn, are regulated by corticotrophin releasing hormone (CRH), a peptide released by CRH neurons in the hypothalamus. There are three levels of regulation for the HPA-axis: diurnal rhythm in basal steroid production, negative feedback regulation by adrenal corticosteroids, and marked increases in steroid production in response to stress [19]. Most effects of glucocorticoids are not immediate, but become apparent after several hours. This fact is of clinical significance, because a delay generally is seen before beneficial effects of glucocorticoid therapy are observed [19].

Glucocorticoids, either naturally occurring (cortisol) or synthetic (e.g. hydrocortisone, prednisone, triamcinolone, dexamethasone), exert a broad range of effects on multiple organ systems and tissues.

7 Rationale for Trial

We hypothesize that a two-week course of Dexamethasone can improve the 6-month functional outcome of patients with symptomatic CSDH by reducing the rate of CSDH-related surgical interventions and the recurrence rate.

The dose and duration of dexamethasone are reflective of other recent studies in the field. Moreover, a maximum of a two-week course of dexamethasone provides the best balance in terms of clinical efficacy and risks.

8 Trial Design

8.1 Statement of design

Dex-CSDH is a pragmatic, multi-centre, double-blind, phase III randomised, placebo controlled trial (n=750) assessing the clinical utility of a tapering 2-week course of dexamethasone following Chronic Subdural Haematoma. It will commence with an internal pilot, the Stage 1 (feasibility study), which will be followed by the Stage 2 (substantive study) if the following progression criteria are met

1. The target recruitment rate is 2 patients per site per month. If there is a >30% shortfall from the recruitment target (i.e. less than 70 patients have been recruited by month 12 of stage 1) without an identifiable and correctable reason it would not be feasible to progress to the main trial.
2. If the loss to follow-up (primary endpoint) exceeds 15% without an identifiable and correctable reason it would not be feasible to progress to the main trial (stage 2) without substantial changes in the study design.
3. No ethical or safety concerns raised by the independent Data Monitoring & Ethics Committee.

8.2 Number of Centres

Stage 1 will take place initially in a limited number of centres in the UK, to ensure feasibility. If successful, stage 2 will follow and will encompass, where possible, all remaining Neurosurgical centres in the UK and the Republic of Ireland.

8.3 Number of Patients

Stage 1 aims to recruit 100 patients within 12 months. If the progression rules are met, Stage 2 will recruit 650 patients within 2 years.

The recruitment rate has been estimated at 2 patients per site per month. On the basis of hospital episode statistics (HES) and data from the ongoing national CSDH audit [17], approximately 60-80 patients with a CSDH are admitted in a medium-sized neurosurgical unit each year. Hence, the estimated recruitment rate is feasible.

8.4 Patients Trial duration

Patients will be monitored whilst in the acute Neurosurgical unit and followed up for a period of 6 months post recruitment.

8.5 Trial objectives

8.5.1 <u>Primary objective</u>:

The primary objective is to detect an 8% absolute difference in the rate of favourable outcome at 6 months between the two arms of the trial with a power of 81-92% and a 2-sided significance of 5%.

8.5.2 Secondary objectives:

- Compare the long-term clinical effectiveness of dexamethasone versus placebo (6 months follow-up period).
- Compare the adverse events and complications between the two arms.
- Undertake a detailed economic evaluation.

8.5.3 Exploratory objectives

- Asses the biological action of dexamethasone with CSDH fluid and blood analysis
- Assess the role of dexamethasone in cerebral perfusion and swelling in CSDH.

8.6 Trial Outcome Measures

8.6.1 Primary outcome measure

The primary outcome measure will be the modified Rankin Scale (mRS) at 6 months post-randomisation.

8.6.2 Secondary outcome measure

The secondary outcome measures are:

1. Number of CSDH-related surgical interventions undertaken during the index admission.
2. Number of CSDH-related surgical interventions undertaken during subsequent admissions in the follow-up period.
3. Glasgow Coma Scale (GCS) at discharge from NSU and at 6 months.
4. mRS score at discharge from NSU and at 3 months.
5. Barthel Index at discharge from NSU, 3 months and 6 months.
6. Mortality (30-day and 6 months).
7. EuroQOL (EQ-5D) at discharge from NSU, 3 months and 6 months.
8. Length of stay in NSU.
9. Discharge destination from NSU.
10. Length of stay in secondary care.
11. Health-economic analysis.
12. Adverse Events.

8.6.3 Economic Evaluation

An economic analysis will be conducted alongside the trial. Costs will be estimated from the viewpoint of the NHS and personal social services (PSS). Resources associated with provision of dexamethasone will thereby be monitored along with any surgical operation(s) to evacuate the CSDH, length of stay (in NSU and the total for the original hospital stay) and any further hospital admissions /surgical procedures e.g. for recurrence of the CSDH. Additionally, the level of informal care will also be monitored to estimate the opportunity cost for both family/friends carers and patients.

8.6.4 Exploratory outcome measures

To assess the mechanism of action of dexamethasone we will be collecting CSDH fluid and blood samples on selected patients who undergo surgery as part of their standard clinical care. Analysis of inflammatory biomarkers will be performed on the blood and CSDH fluid and compared between the dexamethasone and placebo patients.

A select group of patients in the sub-study will also have MRI scans pre- and post-operatively. This is intended to correlate with the biochemistry data and provide additional information on the mechanism of action of dexamethasone with regard to cerebral perfusion and oedema.

TCD will also be used in a sub-set of patients to collect a bed-side measure of cerebral blood flow to assess whether this can be used to predict recovery and recurrence from CSDH.

9 Selection and withdrawal of patients

The trial aims to run in parallel to standard clinical care. The only difference between the trial pathway and the standard NHS pathway is the addition of a two-week course of either dexamethasone or placebo.

Patients usually present to their local Emergency Department where a CT head scan is undertaken in order to establish the diagnosis. Patients with symptoms attributable to a CSDH are referred and admitted to a NSU. Upon admission to the NSU, they are assessed by the on-call neurosurgical team. The decision for surgery or active monitoring is made on an individual patient basis by the admitting clinical team in conjunction with the patient and their families. This decision usually depends on the severity of the symptoms and/or progression of symptoms. Whether the patient is managed surgically or conservatively, they can be recruited to the trial and trial medication given in combination with their other clinical management. All patients are monitored for approximately a week in the NSU. If their symptoms improve, they may be discharged home or transferred to their local hospital if they need rehabilitation. If their symptoms do not improve or deteriorate, they usually have a repeat CT head scan and a decision for surgery is again made on an individual patient basis by the clinical team.

Screening of patients to determine eligibility for participation in the trial will be undertaken by the neurosurgical team upon admission to the NSU according to the following inclusion / exclusion criteria.

9.1 Inclusion Criteria

1. Adult patients aged 18 years or older.
2. Symptomatic CSDH confirmed on cranial imaging (e.g. CT/MRI - predominantly hypodense or isodense crescentic collection along the cerebral convexity on CT).
3. Patient or Legal Representative is willing and able to provide informed consent or in the absence of a legal representative, an Independent Healthcare Professional provides authorisation for patient enrolment.

9.2 Exclusion Criteria

The presence of any of the following will preclude patient inclusion:

1. Patients with conditions where steroids are clearly contra-indicated
2. Patients who are on (or within 1 month of) regular oral or intravenous glucocorticoid steroids*.
3. Previous enrolment in this trial for a prior episode
4. Time interval from the time of admission to NSU to first dose of trial medication exceeds 72 hours.
5. CSDH in the presence of a cerebrospinal fluid (CSF) shunt.

6. Severe lactose intolerance or any known hypersensitivity to dexamethasone or any of the IMP (investigational medicinal product) excipients.
7. Patients with a previous history of psychotic disorders
8. Unwillingness to take products containing gelatin
9. Concurrent enrolment in any other trial of an investigational medicinal product

*Patients on topical or inhaled steroids are allowed to be recruited into the trial, as are patients who have had 1 intra-operative dose of dexamethasone for anti-emesis.

Examples of glucocorticoid steroids: dexamethasone, prednisolone, hydrocortisone
Example of adrenocorticoid steroid: fludrocortisone

9.2.1 Exclusion criteria for biochemical sub-study:

Patients will be excluded from recruitment to the biochemical part of the study if they have any of the following;

- Active malignancy
- On immunosuppressive drug therapy

9.2.2 Exclusion criteria for MRI sub-study:

Patients will be excluded from recruitment to the MRI part of the study if they have any of the following;

- Renal dysfunction
- Pacemaker or any metal implants

9.3 Treatment Assignment and Randomisation Number

Patients will be randomly assigned to either the control or intervention group with a 1:1 allocation as per a computer generated randomisation schedule stratified by site using permuted blocks of random sizes. An Interactive Web-based Response System (IWRS) will be used for allocating treatment packs to individual patients once confirmation of inclusion criteria being met is confirmed.

9.4 Method of Blinding:

Capsules and packaging for both active and placebo arms will be identical in appearance at the point of issue to patients.

It is estimated that less than 10% of eligible patients will have (or develop during the trial) swallowing difficulties, making oral IMP administration difficult or impossible/unsafe. To ensure the trial can proceed in as representative a population as possible, a pragmatic and cost-effective approach to dosing IMP in this cohort is proposed.

The strategy for managing IMP administration in patients with swallowing difficulties has been developed after discussion and advice from the MHRA. The blinded capsules may, with investigator and local pharmacy approval be opened at the point of administration by ward nursing staff and the contents dispersed in water, for administration either via the oral route or a nasogastric tube if one has been inserted during the routine course of care.

If this scenario occurs, the administering nurse, NHS site pharmacy, and potentially the trial patient will no longer be blinded, because the active dexamethasone is in tablet form that has been over-encapsulated, so may require crushing before dispersal in 15-

30 mls water for nasogastric administration (administer via a nasogastric tube), and the placebo will be in powder form.

To maintain blinding of the neurosurgeons, the presence of tablets being inside the opened capsule should not be documented in the medical notes, but referred to in generic terms eg "Capsule contents were mixed with water for NG administration" so that blinding is maintained.

Every effort must be made to maintain patient blinding when NG administration is used, by the patient not seeing the capsules being opened. Should, despite these efforts, the patient discover their treatment, they should be asked to not disclose their treatment allocation to any of the other medical personnel they interact with, eg surgeons, etc. The research staff and outcome assessors will remain blinded.

There are also clinical aspects that could potentially unblind trial team members to treatments allocated.

Patients receiving dexamethasone will be more likely to have higher blood glucose levels compared to those receiving placebo. This may provide an indication-but not proof- that a patient is in the active arm. Concealment of glucose measurements will be difficult as clinical action may be required. However, any decision about surgery is made based on the severity of symptoms and/or progression of symptoms. It is not conceivable that a hint that a patient is in the active arm would influence any decisions about operative or non-operative management, especially because the use of steroids is limited in the UK. Overall, we believe that blinding of the clinical staff (neurosurgeons) making decisions about surgery will be maintained throughout the trial.

9.5 Emergency Unblinding

Emergency unblinding will be managed according to the emergency unblinding procedure using the IWRS, which will be documented in the IMP Handling manual and accessible by the study team. Emergency unblinding requested by the patient's clinical team will only occur in exceptional circumstances (e.g. need to treat a serious adverse event) when knowledge of the actual treatment is absolutely essential for further management of the patient. In the event of the need for unblinding, the clinical team should contact a named member of the research team to discuss, if it is deemed that there is sufficient time. In the event that the clinician feels unblinding is required urgently, then this will be allowed and should be managed according to the trial SOP.

However, we anticipate that the requests for emergency unblinding by the clinical teams will be minimal, due to the favourable safety profile of dexamethasone which is routinely used for patients with brain tumours.

9.6 Patient withdrawal criteria

Each patient has the right to discontinue their participation in the trial at any time. If an unconscious patient regains capacity and makes a request to be withdrawn from the trial then this is accepted. Incapacitated patients may also be withdrawn from the trial if the consultee requests withdrawal. In addition, the investigator may withdraw the patient from their allocated treatment arm if, subsequent to randomisation, a clinical reason for not providing the drug treatment is discovered. As the trial is on an intention to treat basis, any data collected will remain in the trial and the patient will continue to be followed up unless consent is withdrawn. Patients who have been withdrawn from

the trial will not be replaced as the power calculation for the trial allows for a 15% loss to follow up.

All discontinuations and withdrawals will be documented. If a patient wishes to discontinue, anonymised data collected up until that point will be included in the analysis.

Patients requiring nasogastric administration of IMP who are discharged to another hospital during the course of their trial treatment will be withdrawn from the trial medication. Any data collected will remain in the trial and the patient will continue to completion.

10 Trial Treatments:

The IMP is a two-week tapering course of either dexamethasone or matched placebo.

10.1 Dosage schedules

Table 1.Trial medication administration schedule.

On Day 1 of treatment, the 1st dose should be administered as soon as practicable post-randomisation, with the 2nd dose prescribed as per standard timing. If the timing of randomisation dictates, it is permitted to administer the total Day 1 IMP as a single dose. Day 1 start date must be marked on the trial medication bottle.

In special circumstances (such as patients who are nil by mouth for surgery), where study medication is missed at lunchtime, that day's dose(s) may be taken later as long as it is on the same day. Otherwise, the instructions for missed medication are as shown below.

Trial medication administration schedule

- *Day 1 = day of first dose*
- *Dexamethasone 2mg capsules or matched placebo capsules*
- *Route of administration: Oral or via nasogastric tube (see protocol 9.4)*

- 4 capsules in the morning and 4 at lunchtime for days 1, 2, 3
- 3 capsules in the morning and 3 at lunchtime for days 4, 5, 6
- 2 capsules in the morning and 2 at lunchtime for days 7, 8, 9
- 1 capsules in the morning and 1 at lunchtime for days 10, 11, 12
- 1 capsule once daily for days 13, 14
- Stop

Day 14 is the last day of treatment. Do not take any more capsules.

In the event of missing a dose of medication, these can be taken when remembered, but only up to the time of the next planned dose on the same day.

10.1.1 Route(s) of Administration and Maximum dosage allowed

Oral route or nasogastric tube administration (as required). The maximum dosage is 16mg dexamethasone in a single day (at 8mg twice daily for days 1, 2 & 3). Once daily dosages (days 13 & 14) should preferably be administered in the morning.

10.1.2 Maximum duration of treatment of a patient

The maximum duration of treatment for a patient is 14 days. No overage of trial medication is available, so any lost capsules will be considered as missed doses and must be indicated on the medication diary.

Irrespective of whether or not an operation is undertaken, patients will complete the two-week course of trial medication. Patients may be discharged or transferred to a local hospital prior to the completion of the two-week course; in this case, letters will be provided to the patient and medical and pharmacy teams at the local hospital along with the remaining trial medication to ensure that the course is completed.

The exception to this will be in the event of a patient receiving IMP via the nasogastric route: IMP will be stopped at discharge/ transfer if this is the case.

10.1.3 Comparator product

Placebo is a capsule, visually indistinguishable from the active treatment and containing inactive excipients only.

10.2 Presentation of the drug

Dexamethasone capsules will consist of overencapsulated dexamethasone 2mg tablets. A proprietary brand will be used. The excipients used for backfilling the capsules are used as standard tableting excipients

The excipients used to fill the placebo capsule will be the same as those used for backfilling the active capsule.

The IMP will be supplied in individually numbered patient packs (bottles).

Please refer to the Dex-CSDH IMP Handling Manual for further information.

10.3 Known drug reactions & interaction with other therapies

Drug interactions

- *Hepatic microsomal enzyme inducers:* medicines that induce hepatic enzyme cytochrome P-450 isozyme 3A4 such as Phenobarbital, phenytoin, rifampicin, rifabutin, carbamazepine, primidone and aminogluethimide may reduce the therapeutic efficacy of corticosteroids by increasing the rate of metabolism.

- *Hepatic microsomal enzyme inhibitors:* medicines that inhibit hepatic enzyme cytochrome P-450 isozyme 3A4 such as ketoconazole, ciclosporin or ritonavir may decrease glucocorticoid clearance. A reduction in corticosteroid dose may be needed to reduce the risk of adverse effects.

- *Antidiabetic agents:* corticosteroids may increase blood glucose levels. Patients may need dosage adjustment of any concurrent antidiabetic therapy.

- *Non-steroidal anti-inflammatory drugs (NSAIDs):* concomitant administration may increase the risk of gastrointestinal (GI) ulceration. Aspirin should be used cautiously in conjunction with corticosteroids in patients with hypothrombinaemia. The renal clearance of salicylates is increased by corticosteroids and steroid withdrawal may result in salicylate intoxication. Patients should be observed closely for adverse effects of either medicine.

- *Anticoagulants:* response to anticoagulants may be reduced or less often enhanced by corticosteroids. Close monitoring of the International normalized ratio (INR) or prothrombin time is recommended.

- *Antifungals:* the risk of hypokalaemia may be increased with amphotericin.

- *Cardiac glycosides:* there is a risk of toxicity if hypokalaemia occurs due to corticosteroid treatment.

- *Mifepristone:* the effect of corticosteroids may be reduced for 3-4 days after mifepristone.

- *Vaccines:* live vaccines should not be given to individuals with impaired immune responsiveness. The antibody response to other vaccines may be diminished.

- *Oestrogens:* oestrogens may potentiate the effects of glucocorticoids. The dose of corticosteroid may need to be adjusted if oestrogen therapy is commenced or stopped.

- *Somatropin:* the growth promoting effect may be inhibited.

- *Sympathomimetics:* there is an increased risk of hypokalaemia if high doses of corticosteroids are given with high doses of salbutamol, salmeterol, terbutaline or formoteral.

- *Diuretics:* excessive potassium loss may be experienced if glucocorticoids and potassium-depleting diuretics (such as frusemide and thiazides) or carbonic anhydrase inhibitors (such as acetazolamide) are given together.

- *Antacids:* concurrent use of antacids may decrease absorption of corticosteroids – efficacy may be decreased sufficiently to require dosage adjustments in patients receiving small doses of corticosteroids.

10.4 Dosage modifications

No dose modifications are permitted within this trial.

10.5 Legal status of the drug

The trial is being carried out under a Clinical Trial Authorisation. The drug is therefore only to be used by the named investigators, for the patients specified in this protocol, and within the trial.

In standard use, dexamethasone is a licensed Prescription Only Medicine, used in many therapeutic indications.

10.6 Drug storage and supply

The over-encapsulated dexamethasone 2mg tablets and matched placebo to be stored at room temperature as per labelled instructions.

The IMP capsules will be supplied in individual treatment packs. IMP packs will be labelled with a unique and randomly generated identification number to ensure blinding during routine practice and allow emergency unblinding if required. The Royal Free Hospital (RFH) Pharmaceutical Production Unit MIA (IMP) 11149, will manufacture and provide technical QP certification for all IMP batches produced prior to storage and subsequent distribution to sites.

The batch supply of IMP to site pharmacies will be coordinated using the trial's Interactive Web-based Response System (IWRS).

Investigator sites will be responsible for maintaining accurate records of receipt, handling, storage and dispensing of IMP. It is recognised that, in a trial where a significant proportion of inpatients are expected to be transferred to another (non-trial site) hospital during the course of their treatment, retrieval of used treatment packs and any remaining IMP may be problematic and would incur additional postage and packaging costs. Due to these factors and taking into account the pragmatic nature of this trial, it will not be a requirement that used treatment packs and unused IMP are returned for reconciliation. The treatment pack labelling will be such that a physical record of the pack dispensed is retained on the accountability log (to demonstrate that the correct treatment pack was dispensed). Trial teams will ensure compliance with treatment is documented, using source data which could include the inpatient medication administration record and the trial medication diary as well as performing physical capsule counts during inpatient treatment where possible; they will also be responsible for obtaining information from the patient and/or transfer hospital should the course of treatment not be completed while still an inpatient at the investigator site.

Please refer to the Dex-CSDH IMP Handling Manual for further information.

10.7 Concomitant therapy

Any concomitant therapy clinically required will be permitted, including gastroprotection as per local policy. A list of contraindicated concomitant therapies to be avoided during the trial is detailed in sections 4.3 and 4.5 of the current SmPC for dexamethasone. Only concomitant therapies of interest will be recorded on the concomitant medication log in the CRF. These are as follows;

- Gastroprotection
- Anti-diabetic medication
- Intra-operative dose of dexamethasone

11 Procedures and assessments

11.1 Screening evaluation

Screening will be carried out by a member of the clinical team. A screening log will be completed.

Consent must be taken prior to study randomisation and study drug administration. Study dosing must start within 72 hours of admission. Randomisation can take place before or after initial index surgery has taken place.

11.1.1 Identification of potential patients

All patients who have been admitted to the Neurosurgical Unit (NSU) with a confirmed CSDH may be screened for eligibility. A member of the clinical team will assess potential eligibility of these patients based on the inclusion/exclusion criteria outlined in Section 9.1 and 9.2

11.1.3 Patient screening and consent

Where potential patients fulfil the eligibility criteria, they will be approached by a member of the research team who will provide the patient information sheet and clarify any information from the patient/relatives which may preclude recruitment.

At Cambridge only, patients will also be screened for eligibility for the exploratory sub-study (blood and CSDH fluid biochemistry and imaging). If they are eligible they will be given an additional page in the patient information and consent sheet so that they can consider if they would like to take part in any of the additional sub-studies. If they do not wish to take part in these, it will not affect their recruitment to the main trial.

Wherever possible informed consent will be obtained from the patient, however, due to the nature of the condition, this may not be possible.

Despite lacking capacity, patients with subdural haematomas can still be enrolled in the trial. The process for obtaining consent in this situation is detailed in section 11.1.3.1 below.

11.1.3.1 Patient legal representative available in the hospital

In patients lacking capacity, a legal representative will be sought. If the legal representative is available in the hospital, is contactable, or is due to visit the patient within a reasonable timescale, then they will be provided with information about the trial and asked if they will provide consent for the patient before enrolment. This will take place during their visit to the patient.

11.1.3.2 *Patient legal representative not available in the hospital*

Due to the condition and average age of these patients, there will be those who will have no registered legal representative or where the legal representative is not contactable or able to visit the hospital at short-enough notice to be able to start study medication within 72 hours from admission. In such cases we advocate enrolment would be possible with written agreement from an independent clinician. Although we have allowed a maximum of 72 hours between time of admission to first dose of trial medication, recruitment at the earliest time point will be important as many patients will require urgent surgery. Further to this there is a perceived benefit of early intervention with the trial drug to gain maximum benefit from it. If no legal representative is available for discussion then an independent clinician will be approached. If a legal representative visits the hospital at a later date then the trial will be discussed with them and their consent sought at that time point to continue in the trial.

11.1.3.3 Patients who regain capacity will be informed about the clinical trial and consent to continue will be sought during their in-patient stay and if still lacking capacity on discharge, at their 6 month follow-up appointment (if attended). If at any stage either the legal representative or the patient chose to withhold consent then the patient will be withdrawn from the trial.

11.1.3.4 *Independent Healthcare Professional (IHP) Definition*
For the purposes of the Dex-CSDH trial, the Independent Healthcare Professional (IHP) is defined as:

A person who is not connected with the conduct of the trial, specifically:

a) The sponsor of the trial;
b) A person employed or engaged by or acting under the arrangements with the sponsor, and who undertakes activities connected with the management of the trial;
c) An investigator of the trial or;
d) A health care professional who is a member of the investigators team for the purposes of the trial.

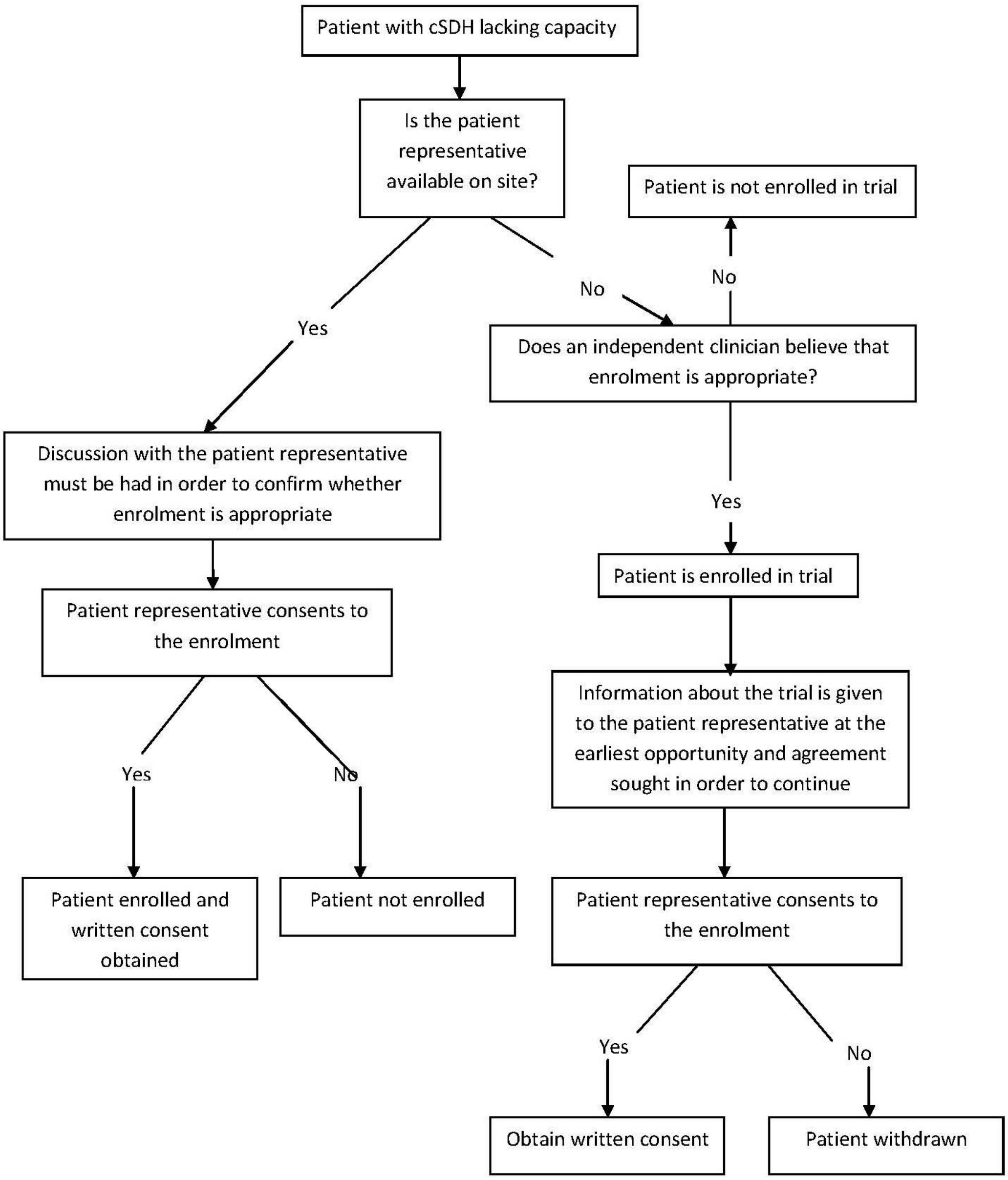

Figure 2. Consent algorithm.

11.1.4 <u>Medical history</u>
Medical history which would preclude eligibility will be obtained from either the patient case notes or after discussion with the patient (or patient's representative) (if applicable).

11.2.5 <u>Patient enrolment/randomisation</u>
When patients pass screening, have been consented, and are enrolled, a unique randomisation number will be allocated to each patient. Once completed, the initial section of the CRF will be faxed, emailed or posted to the Trial Co-ordinating Centre for data entry into the clinical trial database.

All enrolled patients will be offered an optional study wrist band, to be applied before their first dose of medication and worn whilst they are an inpatient. This highlights that they are taking part in a blinded study with either placebo or dexamethasone. This is to help the patient and nursing staff looking after them be aware of the study at all times and to reduce the risk of open label ward dexamethasone stocks being used in error.

Screening forms for patients who are not eligible for the trial will not be faxed for data entry. However, a tally of the patients approached along with the numbers of, and reasons for, screen failures will be kept at each site on a screening log and reported to the Trial Co-ordinating Centre on a monthly basis.

11.2 Baseline assessments

All patients will have a medical history taken and a clinical examination as part of the routine standard of care. The following are to be recorded in part 1 of the case report form (CRF):

- Inclusion/exclusion criteria review
- Informed consent process followed and consent or authorisation for enrolment obtained
- Review of routine lab results

Standard of Care

- Patient medical history (including co-morbidities and relevant medications), and patient demography
- Injury related events - start date of suspected/proven CSDH and/or start of symptoms or suspected injury
- Neurological status
- Imaging modality, date of examination and original images at selected sites for review.

11.3 Exploratory assessment procedures

At Cambridge only, additional data will be collected on CSDH fluid, blood and cranial imaging. Please refer to the DEX-CSDH Laboratory and Imaging Manuals for full details of sample handling and processing.

11.3.1 Subdural fluid sampling:

- During the routine clinical CSDH operation a subdural fluid sample will be collected and stored at 4°C.
- Subdural fluid samples will also be aspirated from the drain at regular time points post-operatively, until the drain is removed.
- The total volume of drainage will be measured at each time point and the samples will be assessed in the same way as intra-operative fluid samples.

11.3.2 Blood sampling:

- One blood sample will be taken when the participant is in theatre for their CSDH operation and stored at 4°c until further analysis.

11.3.3 Transcranial Doppler:

- TCD will be performed at the participant's bedside using a small headset (similar to head phones) with an ultra-sound probe resting on the participant's temples both sides.
- They will also have a small finger probe measuring their BP continuously.
- Monitoring will be set up by a member of research team who will stay with the participant throughout the monitoring period, approximately 30 minutes.
- The following parameters will be recorded:
    - cerebral blood flow velocity
    - pulsatility index

11.3.4 Magnetic Resonance Imaging (MRI)

- MRI will be performed within 72hrs of recruitment and repeated prior to discharge (at least 48 hrs after admission scan and within 14 days of recruitment) and at follow-up (6-12 weeks after recruitment)
- All patients will have had their renal function checked as part of routine clinical care and will only be recruited if this is normal.
- All patients recruited to this sub-study will require intra-venous access for their scan. Most patients will already have this as part of standard clinical care in CSDH but if this is not already in place, then it will be inserted prior to the MRI.
- The imaging protocol will take approximately 50 minutes to complete, and include sequences outlined in the imaging manual.

11.4 Trial assessments

Day 1 to day 14

- IMP administration (in accordance with the dosing schedule)
- AE review
- Medication diary completion
- Review of concomitant medications of interest
- Review of routine lab results
- Collection of CSDH fluid and blood samples for exploratory sub-study participants
- Collection of TCD data for exploratory sub-study participants at 2 time points
    - Before starting study drug
    - 1-7 days after starting treatment
- Collection of MRI data for exploratory sub-study participants
    - Admission (within 72hrs of recruitment)
    - Prior to discharge (within 14 days of recruitment, minimum of 48hrs after previous scan)

Day 14 to discharge from acute neurosurgical unit (if applicable)

- AE review

Day 15 (+/- 1 week)

- Telephone call to assess medication diary
- AE review

Day 30 (+/- 1 week)

- AE review
- Mortality assessment

Discharge from NSU (+/- 1 week) or at death

- AE review
- Review of routine lab results
- Completed CRF faxed to Trial Coordinating Centre
- Mortality assessment
- GCS
- Patient questionnaire (mRS, EQ-5D and Barthel Index) - collected by local site research team

Month 3 (- 4 weeks/+ 8 weeks)

- Patient questionnaire (mRS, EQ-5D and Barthel Index) - collected by central trial co-ordination team
- TCD in exploratory sub-study participants
- MRI (6-12 weeks after recruitment) in exploratory sub-study participants

Month 6 (- 4 weeks/+8 weeks)

- GCS - collected by local site research team if patient attends in clinic
- Patient questionnaire (mRS, EQ-5D, Barthel Index and Health Service Questionnaire) - collected by central trial co-ordination team)
- Mortality assessment

11.4.1 Timing of assessments

Patients will be monitored as per routine clinical practice in the Neurosurgical Unit until discharge and thereafter at approximately 3 and 6 months to score clinical outcome.

Follow up will be by postal questionnaire. However, if after 2 weeks the questionnaire has not been returned then patients will be followed up by telephone. If after a further 4 weeks there is no response then the patient will be deemed as lost to follow up. Where patients attend for a routine clinical follow-up they will be reviewed by a blinded assessor.

Table 2. Schedule of Assessments

	Prior to randomisation (<72 hours from admission)	Randomisation (Preferable, but not essential for this to occur before surgery)	Intra-operative (if applicable)	Day 1 of trial drug (<72 hrs from admission)	Day2-14 of trial drug	Day 15 (+/- 1 week)	Day 30(+/- 1 week)	Discharge from NSU or at death (+/- 1 week)	3 month follow up (-4/ +8wks)	6 month follow up (- 4/ +8wks)
**Eligibility assessment**	X									
**Informed consent**	X							x		If attends OPA
**Randomisation**		X								
**Part 1 of CRF sent to co-ordinating centre**		X								
**IMP Administration**				X	X					
**Review of AEs**		X		X	X	x	X	X		
**Review of concomitant medication**				X	X					
**Telephone call to assess medication diary**						X				
**Completed CRF faxed to Trial Co-ordinating Centre**								X		
**Review of routine lab results**	X			X	X			X		
**GCS**								X		If attends OPA
**mRS**								X	X	X
**Mortality**							X			X
**EQ-5D**								X	X	X
**Barthel Index**								X	X	X
**Health Service Questionnaire**										X

	Prior to randomisation (<72 hours from admission)	Randomisation (Preferable, but not essential for this to occur before surgery)	Intra-operative (if applicable)	Day 1 of trial drug (<72 hrs from admission)	Day2-14 of trial drug	Day 15 (approx)	Day 30	Discharge from NSU or at death	3 month follow up (-4/+8 weeks)	6 month follow up (-4/+8 weeks)
**MRI (sub-study only)**		X (pre-op)			X (post-op)				X (6-12 weeks)	
**TCD (sub-study only)**		x			x				x	
**CSDH fluid (sub-study only)**			x		x					
**Blood sample (sub-study only)**			x							

11.5 Schedule of Assessments

The trial schedule assessments are outlined in Table 2.

The main study CRF will capture core outcomes in CSDH. These will include

- CSDH Recurrence
- Complications
- Functional outcomes
- Radiological outcomes

The assessments for exploratory outcomes will take place at Cambridge only and will be captured on a separate sub-study CRF.

11.6 Trial restrictions

None

12 Assessment of Safety

12.1 Definitions

12.1.1 Adverse event (AE)

Any untoward medical occurrence in a patient or clinical trial patient administered a medicinal product and which does not necessarily have a causal relationship with this treatment.
An adverse event can therefore be any unfavourable and unintended sign (including an abnormal laboratory finding), symptom, or disease temporally associated with the use of an investigational medicinal product, whether or not considered related to the investigational medicinal product.

Recording of adverse events must start from the point of Informed Consent regardless of whether a patient has yet received a medicinal product.

However due to the pathology of the clinical condition where the patients will regularly be monitored in the intensive care environment or on the neurosurgical wards, it is not practicable to record all adverse events. Therefore only adverse events of special interest (AESIs) will be recorded and **reportable** serious adverse events (SAEs) will be reported in accordance with this protocol. (Please see Section 12.3 for details of SAEs that are exempt from expedited reporting.)

Adverse events of special interest (AESI)
The following should all be recorded on the AESI form in the CRF.

- Hyperglycaemia necessitating treatment
- Hyperglycaemia necessitating stopping of trial medication
- New onset diabetes necessitating on-going medical treatment at day 30 follow-up
- Hyperosmolar hyperglycaemic state
- New onset psychosis
- Upper gastrointestinal side effects (e.g. heartburn, vomiting)
- Peptic ulceration and gastro-intestinal bleeding

12.1.2 Adverse reaction to an investigational medicinal product (AR)

All untoward and unintended responses to an investigational medicinal product related to any dose administered. All adverse events judged by either the reporting investigator or the sponsor as having a reasonable causal relationship to a medicinal product qualify as adverse reactions. The expression reasonable causal relationship means to convey in general that there is evidence or argument to suggest a causal relationship.

12.1.3 Unexpected adverse events/reaction

An adverse reaction, the nature, or severity of which is not consistent with the applicable reference safety information (RSI).
When the outcome of the adverse reaction is not consistent with the applicable reference safety information (RSI) this adverse reaction should be considered as unexpected.

12.1.4 Serious adverse event or serious adverse reaction (SAE / SAR)

Any untoward medical occurrence or effect that:

- results in death,
- is life-threatening
- requires hospitalization or prolongation of existing inpatients´ hospitalization
- results in persistent or significant disability or incapacity
- is a congenital anomaly or birth defect
- Is an other important medical event

Life-threatening in the definition of a serious adverse event or serious adverse reaction refers to an event in which the patient was at risk of death at the time of event; it does not refer to an event which hypothetically might have caused death if it were more severe.

SAEs exempt from expedited reporting:

The initial index surgery will not be reported as an SAE unless any of the above criteria are met, nor will SAEs be reportable if they are deemed due to complications related to the CSDH (as outlined in Section 12.3). However, all SAEs (both reportable or non-reportable) will be captured on the study CRF (Case Report Form) or SAE recording log in the Investigator Site File, so that all serious adverse event data is captured.

For the purposes of this trial, prolonged hospitalisation due to delayed transfer will not be considered a reportable SAE.

12.1.5 Suspected Unexpected Serious Adverse Reaction (SUSAR)

A serious adverse reaction, the nature and severity of which is not consistent with the information set out in the Reference Safety Information.

12.1.6 Reference Safety Information (RSI)

The information used for assessing whether an adverse reaction is expected. This is contained in the Summary of Product Characteristics (SmPC)

For this trial the Reference Safety Information is: section 4.8 – Undesirable effects, of the Aspen Pharma Trading Limited, Dexamethasone Tablets SmPC that has been approved by the MHRA for use in this trial

12.2 Expected Adverse Reactions/Serious Adverse Reactions (AR /SARs)

All expected Adverse Reactions are listed in the latest version of the reference safety information as specified in the SmPC. This must be used when making a determination as to the expectedness of the adverse reaction. If the adverse reaction meets the criteria for seriousness, this must be reported as per section 12.7.

12.3 Expected Adverse Events/Serious Adverse Events (AE/SAE)

Expected procedural related adverse events (If SAEs these are exempt from expedited reporting)

Due to the nature of the condition and the characteristics of the patient population, affected individuals can often develop surgical and medical complications. In-hospital death can occur in approximately 5% of patients with a CSDH. Adverse events can be best classified in terms of peri-operative, early, intermediate and late. The following adverse events are 'expected':

**Peri-operative**	**Early**	**Intermediate**	**Late**
- Rebleeding into cavity forming acute subdural	- Residual CSDH	- Recollection of CSDH	- Recollection of CSDH
- Tension pneumocephalus	- Expansion of contralateral CSDH	- Wound complications other than infection	- Surgical Site Infections
- Intracerebral haemorrhage	- Seizures	- Surgical Site Infections	- Epilepsy
- Residual CSDH exerting mass effect		- Subdural empyema	
- Seizure		- Epilepsy	
- Neurological worsening			
- Anaesthetic complications			

Perioperative

Washout of the chronic subdural haematoma is normally performed through burr holes and therefore is not always under direct vision. This can lead to complications such as ICH from inadvertent placement of a catheter during assisted washout or attempted division of membranes. It may also lead to incomplete washout, especially if membranes are still intact, and therefore on-going CSDH and mass effect post-operatively. During washout an acute source of bleeding may be agitated and if not recognised then a post-operative acute subdural haematoma can form in the cavity. The brain does not always fill the cavity immediately and therefore before closure the cavity is normally filled with saline to try and eliminate air. If a large amount of air becomes trapped in the cavity then it will lead to pneumocephalus which can be under tension and cause increased pressure and midline shift. Many of the patients under-going this procedure are elderly and may have multiple co-morbidities, therefore they are considered a high anaesthetic risk.

Early

Pneumocephalus can continue to be an issue in the first few days post-operatively. As the brain re-expands to fill the space there is the additional risk of formation or increase in size of a contralateral CSDH. There is also a risk of seizures following evacuation of the CSDH which is more likely in patients with any peri-operative complication such as ASDH, ICH and pneumocephalus.

Intermediate

Often a drain is placed initially to help with reduced risk of recurrence from CSDH, however this is usually removed within 48hrs. In the week following this, there is a risk that the CSDH can recollect. There is also a risk of infection, as with any surgical wound, and if significant then this could become a subdural empyema if there is also a recollection of subdural fluid. Poor wound healing or dehiscence is a risk, particularly as the patients are mostly elderly and will have thin skin which does not heal rapidly. There is an on-going risk of developing epilepsy in the first few weeks post-operatively.

Late
The biggest risk of the longer term is of recollection of the CSDH which may require further surgical treatment. There is also an on-going risk of developing late epilepsy.

12.4 Evaluation of adverse events

The Sponsor expects that adverse events are recorded from the point of informed consent regardless of whether a patient has yet received a medicinal product. Individual adverse events should be evaluated by the investigator. This includes the evaluation of its seriousness, causality and any relationship between the investigational medicinal product(s) and/or concomitant therapy and the adverse event. AEs should only be recorded for the duration of the patient's hospital stay., Assessment of seriousness

Seriousness is assessed against the criteria in section 12.1.4.This defines whether the event is an adverse event, serious adverse event or a serious adverse reaction.

12.4.1 Assessment of causality

Definitely: A causal relationship is clinically/biologically certain. **This is therefore an Adverse Reaction.**

Probable: A causal relationship is clinically / biologically highly plausible and there is a plausible time sequence between onset of the AE and administration of the investigational medicinal product and there is a reasonable response on withdrawal. **This is therefore an Adverse Reaction.**

Possible: A causal relationship is clinically / biologically plausible and there is a plausible time sequence between onset of the AE and administration of the investigational medicinal product. **This is therefore an Adverse Reaction.**

Unlikely: A causal relation is improbable and another documented cause of the AE is most plausible. **This is therefore an Adverse Event.**

Unrelated: A causal relationship can be definitely excluded and another documented cause of the AE is most plausible. **This is therefore an Adverse Event.**

Unlikely and Unrelated causalities are considered NOT to be trial drug related
Definitely, Probable and Possible causalities are considered to be trial drug related

A pre-existing condition must not be recorded as an AE or reported as an SAE unless the condition worsens during the trial and meets the criteria for reporting or recording in the appropriate section of the CRF.

12.4.2 Clinical assessment of severity

Mild: The patient is aware of the event or symptom, but the event or symptom is easily tolerated

Moderate: The patient experiences sufficient discomfort to interfere with or reduce his or her usual level of activity

Severe: Significant impairment of functioning; the patient is unable to carry out

usual activities and / or the patient's life is at risk from the event.

12.4.3 Recording of adverse events

This clinical trial is being conducted in a critical emergency condition. It is important to consider the natural history of the critical medical event affecting each patient enrolled, the expected complications of this event, and the relevance of the complications to the procedures.

All AESIs, including expected systemic and procedure related adverse events, will be assessed by the Investigator and recorded in detail in the medical notes and CRFs. Results of locally performed clinical laboratory tests (full blood count, coagulation, biochemical markers) will also be recorded in the CRF.

AESIs recorded during the trial will be sent to the coordinating centre. At the conclusion of the trial, all AESIs will be subject to statistical analysis, and the analysis and subsequent conclusions will be included in the final trial report. AESIs will be review at Trial Steering Committee (TSC) meetings.

Serious Adverse Events and Serious Adverse Reactions must be reported to the sponsor as detailed in section **12.5.**

12.5 Reporting serious adverse events (SAEs)

Each Principal Investigator must report all reportable serious adverse events to the Chief Investigator, via the Trial Coordinating Centre, using the trial specific SAE form, within 24 hours of their awareness of the event. (For details of SAEs that are exempt from expedited reporting requirements, please see Section 12.3). The Chief Investigator is responsible for ensuring the assessment of all SAEs for expectedness and relatedness is completed and the onward notification of all SAEs to the Sponsor immediately but not more than 24 hours of first notification. The sponsor has to keep detailed records of all SAEs reported to them by the trial team.

The Chief Investigator is also responsible for prompt reporting of all serious adverse event findings to the competent authority in each member stage (eg the MHRA) if they could:

- adversely affect the health of patients
- impact on the conduct of the trial
- alter the risk to benefit ratio of the trial
- alter the competent authority's authorisation to continue the trial in accordance with Directive 2001/20/EC.

The completed SAE form can be faxed or emailed. Details of where to report the SAE's can be found on the Dex-CSDH SAE form and page 2 of the protocol.

12.6 Suspected Unexpected Serious Adverse Reactions (SUSARs)

All suspected adverse reactions related to an investigational medicinal product (the tested IMP and comparators) which occur in the concerned trial, and that are both unexpected and serious (SUSARs) are patient to expedited reporting. Please see section 12.1.6 for the Reference Safety Information to be used in this trial.

12.6.1 Who should report and whom to report to?

The Sponsor delegates the responsibility of notification of SUSARs to the Chief Investigator. The Chief Investigator must report all the relevant safety information previously described, to the:

- Sponsor,
- competent authorities in the member state (eg the MHRA)
- Ethics Committee in the concerned member states

The Chief Investigator shall inform all investigators concerned of relevant information about SUSARs that could adversely affect the safety of patients.

12.6.2 When to report?

12.6.2.1 Fatal or life-threatening SUSARs

All parties listed in 12.6.1 must be notified as soon as possible but no later than **7 calendar days** after the trial team and Sponsor has first knowledge of the minimum criteria for expedited reporting.
In each case relevant follow-up information should be sought and a report completed as soon as possible. It should be communicated to all parties within an additional **8 calendar days**.

12.6.2.2 Non fatal and non life-threatening SUSARs

All other SUSARs and safety issues must be reported to all parties listed in 12.6.1 as soon as possible but no later than **15 calendar days** after first knowledge of the minimum criteria for expedited reporting. Further relevant follow-up information should be given as soon as possible.

12.6.3 How to report?

12.6.3.1 Minimum criteria for initial expedited reporting of SUSARs

Information on the final description and evaluation of an adverse reaction report may not be available within the required time frames for reporting. For regulatory purposes, initial expedited reports should be submitted within the time limits as soon as the minimum following criteria are met:
a. a suspected investigational medicinal product,
b. an identifiable patient (e.g. trial patient code number),
c. an adverse event assessed as serious and unexpected, and for which there is a reasonable suspected causal relationship,
d. an identifiable reporting source,

and, when available and applicable:
e. a unique clinical trial identification (EudraCT number or in case of non-European Community trial's the sponsor's protocol code number).
f. a unique case identification (i.e. sponsor's case identification number).

12.6.3.2 Follow-up reports of SUSARs

In case of incomplete information at the time of initial reporting, all the appropriate information for an adequate analysis of causality should be actively sought from the reporter or other available sources. Further available relevant information should be reported as follow-up reports.
In certain cases, it may be appropriate to conduct follow-up of the long-term outcome of a particular reaction.

12.6.3.3 Format of the SUSARs reports

Electronic reporting is the expected method for expedited reporting of SUSARs to the competent authority. The format and content as defined by the competent authority should be adhered to.

12.7 Pregnancy Reporting

All pregnancies within the trial should be reported to the Chief Investigator and the Sponsor using the relevant Pregnancy Reporting Form within 24 hours of notification. Pregnancy reporting would stop 3 months after the patient's last dose of IMP for example

Pregnancy is not considered an AE unless a negative or consequential outcome is recorded for the mother or child/fetus. If the outcome meets the serious criteria, this would be considered an SAE.

13 Toxicity – Emergency Procedures

In the event of suspected toxicity, the trial drug will be withdrawn. In the event of emergency unblinding, this will be managed by the appropriate SOP.

14 Evaluation of Results (Definitions and response/evaluation of outcome measures)

The unblinded data will be presented to the Data Monitoring Committee, who will meet on a regular basis throughout the trial. The DMC will then prepare a report for the Trial Steering Committee who will provide overall supervision of the trial.

14.1 Response criteria

14.1.1 Survival

These will be measured from the date of randomisation to the 6 month follow up and will be reported for all deaths due to all causes. The cause of death is to be recorded in all instances.

14.1.2 Quality of life

Quality of life will be assessed by means of the EQ-5D questionnaire to generate quality adjusted life years.

15 Storage and Analysis of Samples

Subdural fluid and blood samples will be collected from patients intra-operatively and post-operatively and stored securely for exploratory analyses. Further details on the processing, labelling, handling, storage of these samples can be found in the DEX-CSDH Laboratory Manual

16 Statistics

16.1 Statistical methods

Analysis will be performed on an "intention-to-treat" basis. The primary endpoint is the modified Rankin Scale at 6 months which is then dichotomised to favourable (0-3) vs unfavourable (4-6). The primary analysis will estimate the absolute difference between

the two treatment arms in the proportions achieving a favourable outcome. A normal approximation will be used to produce 95% confidence interval and a 2-sided p-value testing the null hypothesis of no difference. Secondary analysis will include a proportional odds logistic regression of the original mRS score adjusting for baseline covariates (age, GCS).

Further secondary endpoints will be summarized using appropriate techniques according to whether the variable is binary, categorical, continuous or time-to-event. Categorical and binary variables will be summarized using bar charts, frequency tables and comparisons made using logistic regression. Continuous variables will be summarized, broken down by treatment arm, using Box plots, mean, median, SD, max, min and compared using linear regression. Time-to-event variables will be summarized using Kaplan-Meier plots, and compared using the log-rank test.

An economic analysis will be performed to included formal and informal care costs and resources. Appropriate unit costs will be assigned to each item of resource use for a standard price year. The mean incremental cost for those allocated to dexamethasone compared to placebo intervention over the 6 month trial period will be then be estimated.

The economic analysis will enable both the incremental cost and effect associated with dexamethasone compared to placebo intervention to be estimated, as part of the trial analysis. Assuming dominance does not occur (where one option is estimated to be more effective and less costly that the other option) the incremental cost-effectiveness ratio associated with dexamethasone will be estimated and assessed in relation to a range of cost-effectiveness thresholds e.g. £20,000 - £30,000 per QALY is recommended by NICE. The associated level of uncertainty will also be characterised by estimating cost-effectiveness acceptability curves. Sensitivity analysis will also be undertaken to assess the robustness of conclusions to change in key assumptions. In-line with the outcome analysis all analysis will initially be conducted on an intention-to-treat basis.

16.2 Interim analyses

An interim analysis (blinded to all except the IDMC: Independent Data Monitoring Committee) will be performed after an appropriate number of patients have observed 6-month follow-up, in order to confirm the sample size. The TSC, IDMC and statistical team will agree jointly on the most appropriate timing of this interim analysis, taking into account the case mix and parameters the IDMC wishes to estimate. If the sample size needs to be revised, we are able to incorporate the uncertainly in absolute favourable outcomes rates (80% to 85%) in order to achieve an acceptable conditional power as determined by an Independent Data Monitoring Committee (IDMC). If sample size adjustment is necessary, the final analysis will adjust for the inflated type 1 error rate. The primary purpose of the pilot is to assess recruitment rates rather than to make sample size adjustments.

16.3 Number of Patients to be enrolled

750 patients will be enrolled in the trial (100 patients during Stage 1 and 650 patients during Stage 2). The recruitment rate has been estimated at 2 patients per site per month. On the basis of hospital episode statistics (HES) and data from the ongoing national CSDH audit [4], approximately 60-80 patients with a CSDH are admitted in a medium-sized neurosurgical unit each year. Hence, the estimated recruitment rate is feasible.

An 8% increase in the rate of favourable outcome (mRS 0-3) at 6 months is a plausible and clinically important treatment effect [7-11]. Assuming a favourable outcome rate of 80%-85% in the control group [7, 12] and using a 2-sided test at the 5% significance level, a sample size of 750 patients (allowing for a 15% loss to follow-up) will enable us to detect an 8% absolute difference in the rate of favourable outcome with a power of 81%-92%.

16.4 Criteria for the premature termination of the trial

There are no defined criteria for the premature discontinuation of the trial. However the IDMC and TSC will make recommendations on the discontinuation of the trial following review of the ongoing patient safety and efficacy data presented at regular scheduled meetings.

16.5 Procedure to account for missing or spurious patient data

For the primary analysis missing data will be assumed to be missing at random. A sensitivity analysis will be carried out by performing a complete case analysis. As the relevant covariates need to be recorded before the patient can be randomised, we aim to have minimal missing baseline data. There is also an excellent track record for UK-led neurosurgical studies in achieving extremely high rates for follow-up (STICH, STICH II and RESCUEicp studies).

16.6 Definition of the end of the trial

The end of the trial is the date of the last expected 6 month follow-up questionnaire completed for the last-recruited trial patient.

17 Data handling and record keeping

17.1 CRF

All data will be entered into a Case Report Form (CRF) which will be anonymised. All trial data in the CRF must be extracted from and be consistent with the relevant source documents. The CRFs must be completed, dated and signed by the investigator or designee in a timely manner. It remains the responsibility of the investigator for the timing, completeness, legibility and accuracy of the CRF pages. The CRF will be accessible to trial coordinators, data managers, the investigators, Clinical Trial Monitors, Auditors and Inspectors as required.

The patient demographic data sheet will be completed and filed securely separate from the CRFs. This data is required to enable the coordinating site to contact the patients for the 3 and 6 month follow up. This sheet will be destroyed at the end of the trial.

Participating sites

Completed copies of the CRFs should be posted (or faxed or scanned and emailed) to the trial coordination centre within a reasonable timeframe of the pages being completed. The patient demographic sheet will be posted (or scanned and emailed to the CTC's secure nhs.net email or via other alternative secure method) for the purpose of the 3 and 6 month follow ups.

The investigator will retain the original of each completed CRF at site. The investigator will also supply the trial coordination centre with any required, anonymised background information from the medical records as required.

The investigators must ensure that the CRFs and other trial related documentation is sent to the trial coordination centre containing no patient identifiable data.

All CRF pages must be clear, legible and completed in black ink. Any errors should be crossed with a single stroke so that the original entry can still be seen. Corrections should be inserted and the change dated and initialled by the investigator or designee. If it is not clear why the change has been made, an explanation should be written next to the change. Typing correction fluid must not be used.

17.2 Source Data

To enable peer review, monitoring, audit and/or inspection, the investigator must agree to keep records of all participating patients (sufficient information to link records eg CRFs, hospital records, and samples and all original signed Informed Consent Forms. In this trial the following documentation will be considered as source data:

- Patient medical notes, electronic and/or paper as applicable
- Screening Logs
- Informed Consent Forms
- Questionnaires
- Medication diary sheets
- Source data worksheets will be provided to sites to assist them in documentation of medical history, concomitant medications, and adverse events and adverse reactions. The data listed on these worksheets may or may not be source-data depending on whether this is the first point of recording.

17.3 Data Protection & Patient Confidentiality

All investigators and trial site staff involved in this trial must comply with the requirements of the Data Protection Act 1998 and Trust Policy with regards to the collection, storage, processing and disclosure of personal information and will uphold the Act’s core principles.

18 Trial Steering Committee and Data Monitoring and Ethics Committee

The TSC will provide overall supervision with respect to the conduct of the trial. The independent chair of the TC will be Professor Tony Bell (St George’s, University of London). Full details of the TSC membership and remit can be found in the TSC charter.

The ethical and safety aspects of the trial will be overseen by an independent DMC which will be chaired by Professor Martin Smith (The National Hospital for Neurology and Neurosurgery, London). IDMC meetings will be timed so that reports can be fed into the TSC meetings. Full details of the IDMC membership and remit can be found in the IDMC Charter.

19 Ethical & Regulatory considerations

19.1 Consent

The informed consent form must be approved by the Ethics Committee and must be in compliance with GCP, local regulatory requirements and legal requirements. The investigator must ensure that each trial patient, or his/her legally acceptable representative, is fully informed about the nature and objectives of the trial and possible risks associated with their participation.

Informed consent will be obtained following the procedure outlined in section 11.1.3. The investigator will retain the original of each patients signed informed consent forms in the Investigator Site File (ISF).

Should a patient require a verbal translation of the trial documentation by a locally approved interpreter/translator, it is the responsibility of the individual investigator to use locally approved translators. All trial documentation in a different language (other than English), including the translation and back translation of documents must be reviewed and approved by the Sponsor prior to use. All sections of the approved documents must appear in the translation. The translated version must be appropriately dated and version controlled.

Any new information which becomes available, which might affect the patient's willingness to continue participating in the trial will be communicated to the patient and/or their legally acceptable representative as soon as possible.

19.2 Ethical committee review

Before the start of the trial or implementation of any amendment we will obtain approval of the trial protocol, protocol amendments, informed consent forms and other relevant documents (e.g. Patient Information Leaflets and Consent Forms) from the EC. All correspondence with the EC will be retained in the Trial Master File/Investigator Site File.

Annual reports will be submitted to the EC in accordance with national requirements. It is the Chief Investigator's responsibility to produce the annual reports as required.

19.3 Regulatory Compliance

The trial will not commence until a Clinical Trial Authorisation (CTA) is obtained from the competent authority in the member stage (eg the MHRA). The protocol and trial conduct will comply with the Medicines for Human Use (Clinical Trials) Regulations 2004 and any relevant amendments.

Development Safety Update Reports (DSURs)/ Annual Safety Reports will be submitted to the competent authority in accordance with national requirements. It is the Chief Investigators responsibility to produce the annual reports as required.

19.4 Protocol Amendments

Protocol amendments must be reviewed and agreement received from the Sponsor and the funding body for all proposed amendments prior to submission to the EC and/or the competent authority

The only circumstance in which an amendment may be initiated prior to EC and/or competent authority approval is where the change is necessary to eliminate apparent, immediate risks to the patients (Urgent Safety Measures). In this case, accrual of new patients will be halted until the EC and/or competent authority approval has been obtained.

19.5 Peer Review

The trial proposal has been peer-reviewed and is supported by the Academic Committee of the Society of British Neurological Surgeons, the Age and Ageing National Specialty Group of the NIHR CCRN and the British Neurosurgical Trainee Research Collaborative. The support of the UK Neurosurgical Research Network will allow us to roll-out the substantive trial across the NHS. It has also been through the NIHR peer review process as a requirement of the HTA award.

19.6 Declaration of Helsinki and Good Clinical Practice

The trial will be performed in accordance with the spirit and the letter of the declaration of Helsinki, the conditions and principles of Good Clinical Practice, the protocol and applicable local regulatory requirements and laws.

19.7 GCP Training

All trial staff must hold evidence of appropriate GCP training or undergo GCP training prior to undertaking any responsibilities on this trial. This training should be updated in accordance with your Trust's policy.

20 Sponsorship, Financial and Insurance

The trial is sponsored by Cambridge University Hospitals NHS Foundation Trust and University of Cambridge. The trial is funded by the National Institute for Health Research Health Technology Assessment board (NIHR HTA).

Cambridge University Hospitals NHS Foundation Trust, as a member of the NHS Clinical Negligence Scheme for Trusts, will accept full financial liability for harm caused to patients in the clinical trial caused through the negligence of its employees and honorary contract holders. There are no specific arrangements for compensation should a patient be harmed through participation in the trial, but no-one has acted negligently.

The University of Cambridge will arrange insurance for negligent harm caused as a result of protocol design and for non-negligent harm arising through participation in the clinical trial.

21 Monitoring, Audit & Inspection

The investigator must make all trial documentation and related records available should an MHRA Inspection occur. Should a monitoring visit or audit be requested, the investigator must make the trial documentation and source data available to the Sponsor's representative. All patient data must be handled and treated confidentially.

The Sponsor's monitoring frequency will be determined by an initial risk assessment performed prior to the start of the trial. A detailed monitoring plan will be generated detailing the frequency and scope of the monitoring for the trial. Throughout the course of the trial, the risk assessment will be reviewed and the monitoring frequency adjusted as necessary.

Remote monitoring will be conducted for all participating sites. The scope and frequency of the monitoring will be determined by the risk assessment and detailed in the Monitoring Plan for the trial.

22 Protocol Compliance and Breaches of GCP

Prospective, planned deviations or waivers to the protocol are not allowed under the UK regulations on Clinical Trials and must not be used.

Protocol violations, deviations, non-compliances, or breaches are departures from the approved protocol. They can happen at any time, but are not planned. They must be adequately documented on the relevant forms and reported to the Chief Investigator and Sponsor immediately.

Regarding missed study medication, only deviations deemed to be either clinically significant or where the trial site staff caused the error/deviation will be documented as protocol non-compliance. Where patients refused or missed trial medication at their own volition, this will be documented in the medication record but not reported as a protocol non-compliance.

Deviations from the protocol which are found to occur constantly again and again will not be accepted and will require immediate action and could potentially be classified as a serious breach.

Any potential/suspected serious breaches of GCP must be reported immediately to the Sponsor without any delay.

23 Publications policy

Ownership of the data arising from this trial resides with the trial team. On completion of the trial the data will be analysed and tabulated and a Final Trial Report (FSR) will be prepared.

We intend to disseminate the findings of the Dex-CSDH trial via peer-reviewed journals and presentations at national and international meetings. In addition to meetings orientated around neurosurgery, we will target conferences organised for the different health professionals who care for patients with chronic subdural haematoma, including those in emergency medicine for the elderly and general practice. We will publish the results of the trial on the EudraCT website.

Research findings will be disseminated to relevant service user groups and charities (eg Age UK and Headway) through newsletters, website posts and public presentations. The Dex-CSDH trial website will also include dedicated pages for members of the public. We will present the trial in open days organised by hospitals participating in the trial where members of the public are invited to find out about ongoing research. We will also give talks/presentations in meetings of local/regional relevant service user groups and charities (Age UK and Headway local branches).

References

1. Wada T, Kuroda K, Yoshida Y, Ogasawara K, Ogawa A, & Endo S. (2006). Local elevation of the anti-inflammatory interleukin-10 in the pathogenesis of chronic subdural hematoma. Neurosurgical Review, 29(3), 242–5.
2. Hong HJ, Kim YJ, Yi HJ, Ko Y, Oh SJ & Kim JM. (2009). Role of angiogenic growth factors and inflammatory cytokine on recurrence of chronic subdural hematoma. Surgical Neurology, 71(2), 161–165; discussion 165–166.
3. Stanisic M, Aasen AO, Pripp AH, Lindegaard KF, Ramm-Pettersen J, Lyngstadaas SP, Sæhle T. (2012). Local and systemic pro-inflammatory and anti-inflammatory cytokine patterns in patients with chronic subdural hematoma: a prospective study. Inflammation Research : Official Journal of the European Histamine Research Society, 61(8), 845–52.
4. Frati A, Salvati M, Mainiero F, Ippoliti F, Rocchi G, Raco A, Delfini R. (2004). Inflammation markers and risk factors for recurrence in 35 patients with a posttraumatic chronic subdural hematoma: a prospective study. Journal of Neurosurgery, 100(1), 24–32.
5. Kaplan M, Erol FS, Bozgeyik M. The effectiveness of simple drainage technique in improvement of cerebral blood flow in patients with chronic subdural haemorrhage. Turk Neurosurg. 2007; 17(3): 202-6.
6. Weisenberger H Species differences in the hydrolysis of dexamethasone 21-isonicotinate by serum esterases. *Klin. Wschr* 1972, 50: 665.
7. Houghton E, Grainger L, Teale P The *in vitro* hydrolysis of dexamethasone trimethylacetate in whole blood from the horse and cow. Unpublished report from Horseracing Forensic Laboratory, Newmarket, July 1989. Submitted to WHO by Ciba Geigy, Division Agro, Basle, Switzerland. 1989
8. Coert A, Hoeijmakers M, van Rens P. The *in vitro* hydrolysis of dexamethasone dimethylbutyrate in cows plasma. Unpublished subject report no. 10 d.d. 3-2-1988 from IntervetBoxmeer. Submitted to WHO by BoehringerIngelheimVetmedica GmbH, Ingelheim, Germany, 1988
9. Santarius T, Kirkpatrick PJ, Ganesan D, Chia HL, Jalloh I, Smielewski P, Richards HK, Marcus H, Parker RA, Price SJ, Kirollos RW, Pickard JD, Hutchinson PJ. Use of drains versus no drains after burr-hole evacuation of chronic subdural haematoma: a randomised controlled trial. Lancet. 2009 Sep28;**374**(9695): 1067-73. Doi: 10.1016/S0140-6736(09)61115-6.
10. Santarius T, Kirkpatrick PJ, Kolias AG, Hutchinson PJ. Working toward rational and evidence-based treatment of chronic subdural hematoma.ClinNeurosurg. 2010; **57**:112-22.Review. PubMed PMID: 21280503.
11. Delgado López PD, Martín Velasco V, CastillaDíez JM, Rodríguez Salazar A, GalachoHarriero AM, FernándezArconada O. Dexamethasone treatment in chronic subdural haematoma. Neurocirugia (Astur). 2009 Aug;**20**(4):346-59.
12. Sun TF, Boet R, Poon WS. Non-surgical primary treatment of chronic subdural haematoma: Preliminary results of using dexamethasone. Br J Neurosurg. 2005 Aug;**19**(4):327-33.
13. Berhauser Pont LM, Dammers R, Schouten JW, Lingsma HF, Dirven CM. Clinical factors associated with outcome in chronic subdural hematoma: a retrospective cohort study of patients on preoperative corticosteroid therapy. Neurosurgery. 2012 Apr; **70**(4): 873-80; discussion 880. Doi: 10.1227/NEU.0b013e31823672ad.
14. Berghauser Pont LM, Dirven CM, Dippel DW, Verweij BH, Dammers R. The role of corticosteroids in the management of chronic subdural hematoma: a systematic review. Eur J Neurol. 2012 Nov;**19**(11):1397-403. Doi: 10.1111/j.1468-1331.2012.03768.x. Epub 2012 May 29. Review.

15. EmichS, Richling B, McCoy MR, Al-Schameri RA, Ling F, Sun L, Wang Y, Hitzl W. The efficacy of dexamethasone on reduction in the reoperation rate of chronic subdural haematoma – the DRESH study: straightforward study protocol for a randomized controlled trial. Trials. 2014 Jan 6;**15**(1):6.
16. Martindale. The Extra Pharmacopoeia.31st edition. Reynolds JEF (ed.). London: Royal Pharmaceutical Society, 1996: 1017-1058.
17. http://apps.who.int/trialsearch
18. Immunomodulating agents. In: American Medical Association Drug Evaluations Annual 1995. Division of Drugs and Toxicology, 1995: 1941-1983.
19. Schimmer BP, Parker KL. Adrenocorticotropic hormone; adrenocortical steroids and their synthetic analogs; inhibitors of the synthesis and actions of adrenocortical hormones. In: Goodman and Gilman's. The pharmacological basis of therapeutics.Eleventh edition.Brunton LL, Lazo JS, Parker KL (eds.). The McGraw-Hill companies Inc. New York. 2006.
20. Kawai S, Ichikawa Y, Homma M. Differences in metabolic properties among cortisol, prednisolone, and dexamethasone in liver and renal diseases: accelerated metabolism of dexamethasone in renal failure. J ClinEndocrinolMetab 1985;848-854.

24 Appendices

24.1 Appendix 1 - Trial Management / Responsibilities

24.1.1 Patient registration/ Randomisation procedure

On completion of the baseline section of the CRF, the forms should be sent to the Trial Coordinating centre either by fax or email in a timely manner in accordance with the CRF completion guideline.

24.1.2 CRF Completion & Data management

All CRFs should be completed in a timely manner. Data management at the Trial Coordinating Centre will be undertaken by the trial coordinating team. The local Principal Investigators will be responsible for overseeing the collection of accurate data at their participating site. The completed CRFs will be signed by either the Principal Investigator or a suitably qualified and delegated member of their trial team. Data management will entail the checking of the CRFs and ensuring that data queries are completed in a timely manner in accordance with the data management plan.

24.1.3 Preparation & submission of amendments

Amendments to the trial will be prepared and submitted to the appropriate authorities, by a member of the Trial Coordinating team. Subsequent approvals will then be disseminated to all sites, prior to implementation.

24.1.4 Preparation and submission of Annual Safety Report/Annual Progress Reports

A member of the Trial Coordinating team will prepare the ASR and APR reports prior to submission.

24.1.5 Trial Monitoring

The frequency, scope and method of monitoring will be determined by the Sponsor’s trial level risk assessment. All sites will be monitored in accordance with the Sponsor’s SOPs.

24.1.6 Data protection/ confidentiality

All investigators and trial site staff involved in this trial must comply with the requirements of the Data Protection Act 1998 and Trust policy with regards to collection, storage, processing and disclosure of personal information and will uphold the Act’s core principles.

24.1.7 Trial documentation & archiving

All trial documentation will be retained in a secure location during the conduct of the trial and following end of trial, archived in accordance with the Sponsors SOPs. Each site will be responsible for archiving their investigator site file and associated trial documentation.

24.2 Appendix 2 – Authorisation of Participating Sites

24.2.1 Required Documentation

Prior to initiating a participating site the following documentation is required;

- Principal Investigator and other key trial team staff CV (signed and dated) and GCP certificate
- Trial REC/HRA approval
- Competent Authority approval (eg MHRA)
- Local R & D approval or equivalent
- Participating Site Agreement executed, including pharmacy participating site agreement
- Patient Information sheets and consent forms on local headed paper
- Protocol signed and dated by PI
- Delegation of authority Log
- Confirmation of randomisation system training
- Trial specific prescription

24.2.2 Procedure for initiating/opening a new site

When all the regulatory paperwork is in place, prior to site opening, an initiation meeting will take place, either face to face or via a teleconference. This will be led by a member of the Trial Coordinating team with as many of the local team present as is practicable. This initiation meeting constitutes training for the trial and it is therefore imperative that all members of the trial team who will be involved in the trial are represented at the meeting. A log of attendees will be completed during the meeting. The presentation slides will be provided to the site in advance of the meeting. A trial initiation form will be completed for each site initiation meeting. Copies of all initiation documentation must be retained in the Investigator Site File.

The sponsor's regulatory green light procedure will be followed. Following the green light, the initial supply of IMP will be ordered for shipment to the site on the authorisation of the coordinating centre coordinator. Following confirmation of receipt of the IMP at site, the site will be opened for recruitment and the randomisation system opened to that site.

24.2.3 Principal Investigator Responsibilities

The Principal Investigator has overall responsibility for the conduct of the trial at the participating site.

In particular, the PI has responsibilities which include (but are not limited to):

- Ensuring the appropriate approvals are sought and obtained
- Continuing oversight of the trial
- Ensuring the trial is conducted according to the protocol
- Ensuring consent is obtained in accordance with the protocol and national requirements
- Ensuring that the ISF is accurately maintained
- Delegation of activities to appropriately trained staff (this must be documented on the delegation of authority log)

- Providing protocol or specialised training to new members of the trial team and ensuring that if tasks are delegated, the member of staff is appropriately trained and qualified
- Appropriate attendance at the initiation meeting
- Ensuring appropriate attendance at the TSC/IDMC meetings if required and ensuring appropriate safety information is made available to the coordinating centre team in advance of the meetings
- Dissemination of important safety or trial related information to all stakeholders at the participating site
- Safety reporting within the timelines and assessment of causality and expectedness of all SAEs

24.3 Appendix 3 - Safety Reporting Flow Chart

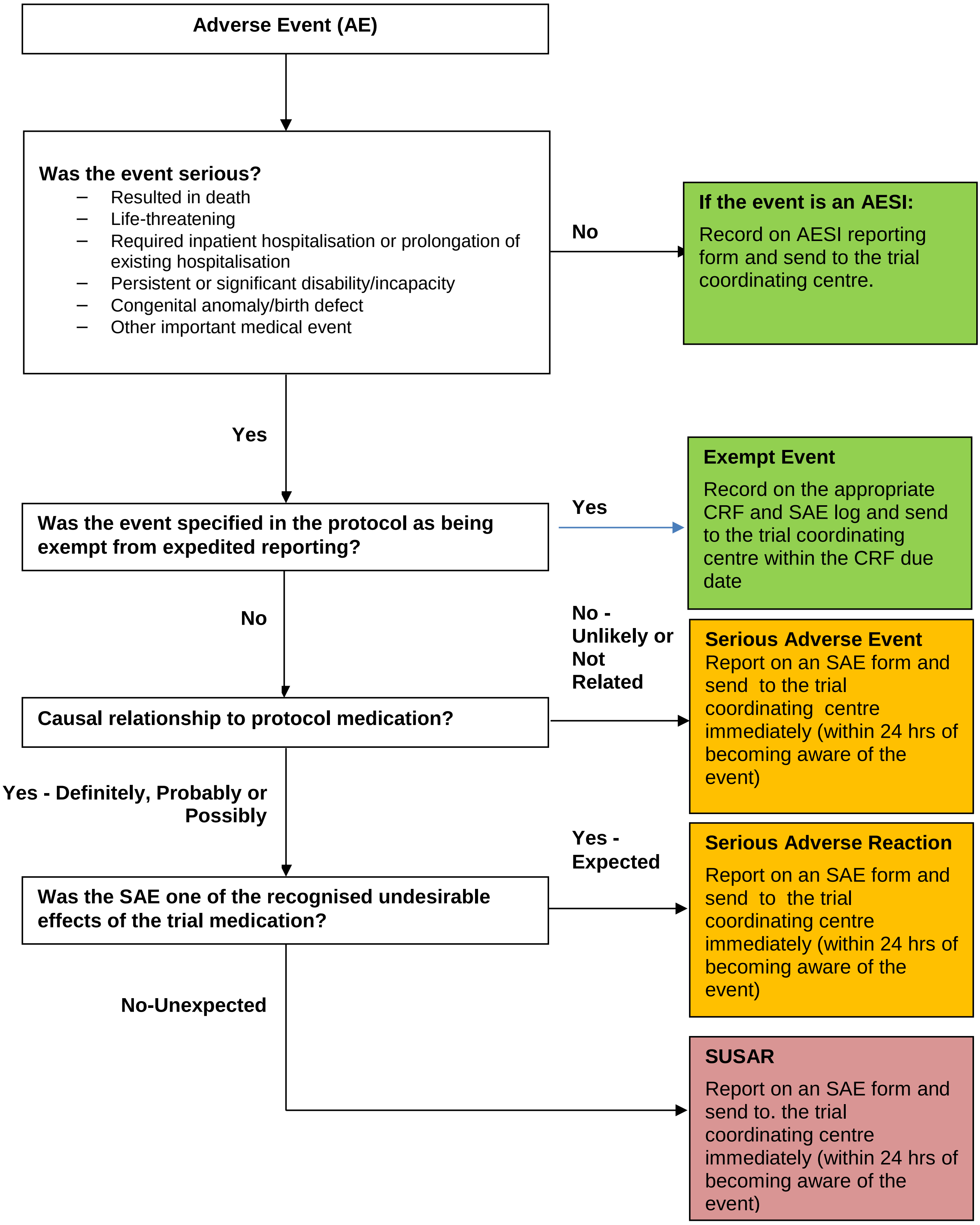

Supplement: Supplementary file 2 — Protocol version 3.0 [file 41598_2019_42087_MOESM2_ESM.pdf]
